# Constructing a novel mitochondrial-related gene signature for evaluating the tumor immune microenvironment and predicting survival in stomach adenocarcinoma

**DOI:** 10.1186/s12967-023-04033-6

**Published:** 2023-03-13

**Authors:** Jingjia Chang, Hao Wu, Jin Wu, Ming Liu, Wentao Zhang, Yanfen Hu, Xintong Zhang, Jing Xu, Li Li, Pengfei Yu, Jianjun Zhu

**Affiliations:** 1 Department of Cell Biology and Medical Genetics, School of Basic Medical Science, Shanxi Medical University, Taiyuan, 030001 China; 2 Department of Pathology, Laboratory of Translational Medicine Research, Deyang People’s Hospital, Deyang, China; 3 Key Laboratory of Tumor Molecular Research of Deyang, Deyang, China; 4 Department of Molecular and Cellular Biology, Roswell Park Comprehensive Cancer Center, Elm and Carlton Streets, Buffalo, NY 14263 USA; 5 Department of Gastrointestinal Surgery, Xijing Hospital, Air Force Military Medical University, Xi’an, 710032 Shaanxi China

**Keywords:** Gastric cancer, Prognostic biomarker, Mitochondrion, Immune cells infiltration, Tumor microenvironment, Immunotherapy, Drug susceptibility

## Abstract

**Background:**

The incidence and mortality of gastric cancer ranks fifth and fourth worldwide among all malignancies, respectively. Accumulating evidences have revealed the close relationship between mitochondrial dysfunction and the initiation and progression of stomach cancer. However, rare prognostic models for mitochondrial-related gene risk have been built up in stomach cancer.

**Methods:**

In current study, the expression and prognostic value of mitochondrial-related genes in stomach adenocarcinoma (STAD) patients were systematically analyzed to establish a mitochondrial-related risk model based on available TCGA and GEO databases. The tumor microenvironment (TME), immune cell infiltration, tumor mutation burden, and drug sensitivity of gastric adenocarcinoma patients were also investigated using R language, GraphPad Prism 8 and online databases.

**Results:**

We established a mitochondrial-related risk prognostic model including NOX4, ALDH3A2, FKBP10 and MAOA and validated its predictive power. This risk model indicated that the immune cell infiltration in high-risk group was significantly different from that in the low-risk group. Besides, the risk score was closely related to TME signature genes and immune checkpoint molecules, suggesting that the immunosuppressive tumor microenvironment might lead to poor prognosis in high-risk groups. Moreover, TIDE analysis demonstrated that combined analysis of risk score and immune score, or stromal score, or microsatellite status could more effectively predict the benefit of immunotherapy in STAD patients with different stratifications. Finally, rapamycin, PD-0325901 and dasatinib were found to be more effective for patients in the high-risk group, whereas AZD7762, CEP-701 and methotrexate were predicted to be more effective for patients in the low-risk group.

**Conclusions:**

Our results suggest that the mitochondrial-related risk model could be a reliable prognostic biomarker for personalized treatment of STAD patients.

**Supplementary Information:**

The online version contains supplementary material available at 10.1186/s12967-023-04033-6.

## Introduction

The incidence (accounting for 5.6% of all cancer cases) and mortality (accounting for 7.7% of all cancer deaths) of gastric cancer (GC) ranks fifth and fourth worldwide among all malignancies, which critically threatens human health. It was estimated that over one million new cases of GC were reported worldwide in 2020 [1]. Gastric adenocarcinomas derived from gastric glandular epithelial cells accounts for more than 90% of all GCs [2]. GC is a multifactorial disease, which is contributed by both environmental and genetic factors [3], such as smoking, family history, Epstein–Barr virus (EBV) infection, alcohol consumption, and diet [4]. Most GCs are diagnosed at late stage of progression due to limited premalignant indications and symptoms [2]. Nowadays, the application of the endoscopic examination largely improved the survival rate of GC patients, and a 30% reduction in GC mortality using endoscopic screening [3]. However, stomach cancer is still one of the most lethal malignant tumors, with a 5 year survival rate of around 20% [5]. Therefore, it is necessary to identify more reliable biomarkers for predicting the prognosis and exploring more potential therapeutic targets in GC.

Accumulating evidences indicated that mitochondria plays essential roles in regulation of cell growth, cell death, and cell metabolism during the whole process of tumor progression [6]. Mitochondria are involved in bioenergetics metabolism, such as ATP production, reactive oxygen species (ROS) production, apoptosis, and calcium homeostasis [7]. Moreover, mitochondrial dysfunction may contribute to the chemoresistance [8]. Therefore, mitochondrial-targeting therapies may be applied for the treatment of GC, including ROS production and elimination, mitochondrial fission and fusion, ATP production, and apoptosis [6, 9, 10]. For instance, nanohybrid-induced oxidative stress triggered mitochondria-mediated autophagy, which inhibited cell growth in cancer cell [11].

Considering that mitochondrial dysfunction was a risk factor for the tumorigenesis of GC, identifying effective mitochondrial-related biomarkers for the prognosis of GC patients should be an encouraging direction of research. Several studies have constructed GC prognosis-related models to predict patient survival [12–14]. However, rare studies have been applied for the establishment of prognostic models for GC associated with mitochondria.

Tumor microenvironment (TME) was mainly composed of the stromal cells, immune cells and cytokines [15]. The components of TME affected the immune cell evasion or inhibition, and drug resistance in malignancies. For example, immune progenitors in the complex microenvironment of the TME were more likely to differentiate into M2 macrophages and Treg cells, but not to play their tumor-inhibiting functions as fully mature immune cells [16]. In addition, the response to immune checkpoint blockade (ICB) was closely related to the constitution of the TME. ICB revived an effective anti-tumor immune response [17]. It was reported that PD-1/PD-L1 inhibitors immunotherapy has an impact on the therapy of patients with advanced gastrointestinal malignancy. Moreover, a study reported that tumors with a higher tumor mutation burden (TMB), had a better immunotherapy response, especially with PD-1/PD-L1 blockade [18]. Thus, to figure out the correlation between the risk score and TME, we explored the TME signatures, the immune cell infiltration in TME, the expression level of immune checkpoints, and the response to immunotherapy.

In summary, a novel mitochondrial-related risk model was constructed using NOX4, FKBP10, ALDH3A2, and MAOA gene set, which could effectively predict the prognosis and immunotherapy responsiveness for patients with STAD. In addition, we estimated the drug sensitivity of STAD patients to 138 drugs, including chemotherapy drugs, immunotherapy drugs, and targeted drugs, et al., and found that patients in high-risk group was more sensitive to rapamycin, PD-0325901 and dasatinib, whereas patients in low-risk group was more sensitive to AZD7762, CEP-701 and methotrexate. Taken together, our mitochondrial-related risk model could be a reliable prognostic biomarker for personalized treatment of STAD patients.

## Methods

### Data collection

RNA-seq data and microsatellite status information for 407 STAD samples were downloaded from the TCGA database (https://www.cancer.gov/tcga). The clinical information was extracted from the UCSC Xena (http://xena.ucsc.edu) [19]. 61 samples were excluded due to incomplete clinical information or survival less than 30 days. In total, 346 samples, comprising 317 tumor samples and 29 healthy samples, were analyzed in the present study. Two validation cohorts, GSE66229 and GSE15459, were applied in the present study (https://www.ncbi.nlm.nih.gov/geo/). In GSE15459, 10 samples were excluded due to survival less than 30 days. Altogether, 300 and 182 samples were analyzed in GSE66229 and GSE15459, respectively. The list of mitochondrial-related genes was collected from MitoCarta 3.0 database (https://www.broadinstitute.org/mitocarta/mitocarta30-inventory-mammalian-mitochondrial-proteins-and-pathways) [20] and the Gene set enrichment analyses (GSEA, http://www.gsea-msigdb.org/gsea/index.jsp) [21, 22] (Additional file 10: Table S1).

### Identification of differentially expressed genes (DEGs)

The “limma” package of R (version 3.5.1) was applied to produce DEGs between normal and tumor samples, or between high-risk and low-risk groups from the training set. |Log (**2**) fold change|> 2 and adjusted *P* < 0.01 were the criteria for defining DEGs. “GdcVolcanoPlot” packages in R were employed to generate volcano map to visualize the DEGs, and a Venn plot was exploited to display the common DEGs in both DEGs groups and mitochondrial-related genes.

### Construction and validation of prognostic mitochondrial-related risk score signature

The mitochondrial-related genes were screened by univariate Cox regression, Lasso regression analysis and multivariable Cox regression analysis to construct a novel prognostic gene signature. Each sample’s risk score was calculated using the following formula:

$${\text{Risk score }} = \, \Sigma {\text{expgenei}} * {\beta i}$$

where expgene, i, and βi represent the expression level of gene, the number of signature genes, and the coefficient index, respectively. In all participated cohorts, the samples were divided into low-risk and high-risk groups based on the risk score (median cut-off value). To analyze the survival conditions for the prognosis signature, the optimized cutoff and the Kaplan–Meier (K–M) survival curve were conducted by R package “survival” and “survminer”. The predictive performance was presented by ROC curve, risk plot and concordance index (C-index). Detailed information for prognostic genes was obtained from The Human Protein Atlas (HPA, https://www.proteinatlas.org/) and National Center for Biotechnology Information (NCBI, https://www.ncbi.nlm.nih.gov/).

### Construction and valuation of nomogram

Risk score and clinical factors including age, gender, T stage, N stage, M stage, tumor stage, family history, H pylori infection, grade, reflux history, and disease types were analyzed using univariate Cox regression analysis to screen the factors significantly related to survival (*P* < 0.1). Then, multivariate COX regression analysis was applied to identify the candidate predictors significantly related to survival (*P* < 0.05). Based on this, nomograms were constructed using these predictors, and scores in nomogram model were assigned for these variables. By adding the scores of the predictors enrolled in nomogram model, the total score of each patient was obtained. Finally, the patient's survival outcome in 1, 3 and 5 years can be calculated using the total score and the probability of survival outcome. ROC curve, calibration curves and decision curve analysis (DCA) were applied to estimate the discrimination and accuracy of the nomogram model.

### Gene Ontology (GO) and Kyoto Encyclopedia of Genes and Genomes (KEGG) analyses

In the present study, R “clusterProfiler”, “org.Hs.eg.db”, “enrichplot” and “ggplot2” package (R version: 3.5.1) were employed to analyze the function of mitochondrial-related DEGs, or the DEGs between high-risk and low-risk groups. Furthermore, adjusted *P* < 0.05 was used to filter the functional candidates.

### Gene set enrichment analyses (GSEA)

Curated sets v7.4 collections were obtained from the Molecular Signatures Database as the target sets with which GSEA was performed by using GSEA 4.2.1 software. The total transcriptome of tumor samples was used for the GSEA, and only gene sets with *P* < 0.001 and FDR,* q* < 0.001 were regarded to be statistically significant.

### Tumor microenvironment

Stromal scores were calculated using the ESTIMATE algorithm by R (version 3.5.1) package “estimate”. The list of TME-related biomarkers was extracted from the Gene set enrichment analyses (GSEA, http://www.gsea-msigdb.org/gsea/index.jsp) [21, 22] (Additional file 10: Table S2–S5).

### Calculation of relative abundance of 22 immune cell subtypes

The abundance of 22 tumor-infiltrating immune cells (TIICs) in STAD samples was calculated using the CIBERSORT algorithm by R package. CIBERSORT is a deconvolution algorithm that can infer 22 kinds of TIICs and harnesses the ability to predict the relative abundance of each immune cell population by calculating the expression of specific marker [23]. The relative abundance of the TIICs between high-risk and low-risk groups was compared using the Wilcox text. The list of immune check points was referenced from a published study [24]. Immune score and tumor purity were also calculated using the ESTIMATE algorithm by R (version 3.5.1) package “estimate”. The list of immune cell signatures was downloaded from TISIDB (http://cis.hku.hk/TISIDB/download.php) [25].

### Prediction of therapeutic sensitivity in patients with different risk scores

The capability of risk score in predicting the response to immunotherapy or 138 drugs for chemotherapies/targeted therapies was explored in the present study. The 50% inhibiting concentration (IC50) values of the 138 drugs were calculated using the “pRRophetic” package of R (version 3.5.1) and the value was normally transformed. The detailed information of 138 drugs was acquired from Genomics of Drug Sensitivity in Cancer (GDSC, https://www.cancerrxgene.org/). The potential response to immunotherapy was inferred by the tumor immune dysfunction and exclusion (TIDE, http://tide.dfci.harvard.edu) score.

### Mutation analysis

The somatic mutation data were downloaded from cBioportal database (https://www.cbioportal.org/) [26, 27]. The R (version 3.5.1) package “maftools” was then used to draw a waterfall plot to illustrate the mutation landscape in STAD patients with the high- and low-risk group and calculate the TMB score for each sample.

### Cell culture and patient sample collection

The normal gastric epithelial cell line GES-1 and human gastric cancer cell lines SGC-7901 and HGC-27 were purchased from the American Type Culture Collection (ATCC, Manassas, VA, USA). Cells were cultured in DMEM medium (Gibco, Thermo Fisher Scientific, Inc., Waltham, MA, USA) supplemented with 10% fetal bovine serum (FBS, Hyclone). Cells were routinely cultured in a humidified atmosphere containg 5% CO_2_ at 37 ℃. A total of 10 fresh tumor and paired adjacent normal tissues from patients with STAD were collected in the First Affiliated Hospital of Shanxi Medical University (Taiyuan, Shanxi, China). All patients provided written informed consent, and this study was approved by the ethics committee of Shanxi Medical University.

### RNA extraction and qRT-PCR assays

RNAs in tissues and cell lines of STAD were extracted with a RNAiso Plus (Takara,Tokyo, Japan) and were reversely transcribed into cDNA usin PrimeScriptTM RT Master Mix (Takara). Quantitative real-time PCR (qRT-PCR) was performed by TB Green ^®^Premix Ex TaqTM II (Takara). β-actin was used as reference genes. The primers were listed in Additional file 10: Table S6.

### Statistical analysis

R (version 3.5.1) and the GraphPad Prism 8 software were applied for statistical analysis. A Student’s *t*-test was used to analyze the expression and the distribution of risk score, stromal score, immune score, tumor purity and TMB in different groups. Chi-square test is applied to evaluate the difference in immunotherapy response, status of top 5 mutant genes and clinical factors in different groups. The correlation was evaluated using the Spearman method. C-index was used to estimate the predictive power of age and risk score to OS.* P* < 0.05 was defined as statistically significant.

## Results

### Identification of DEGs related to mitochondrion and functional enrichment analysis in STAD

**Fig. 1:**
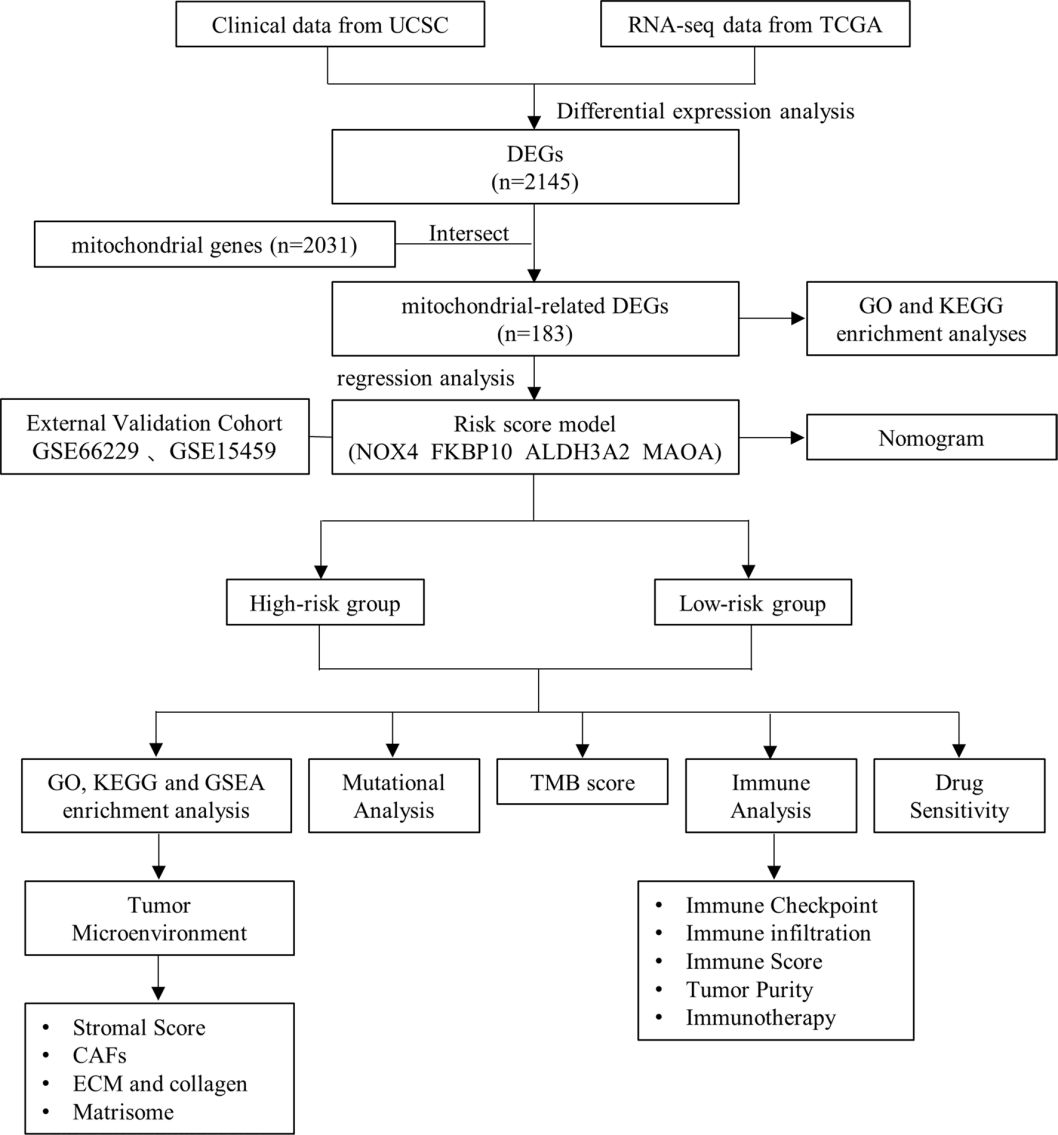
Workflow diagram. The flowchart graph of this study

**Fig. 2:**
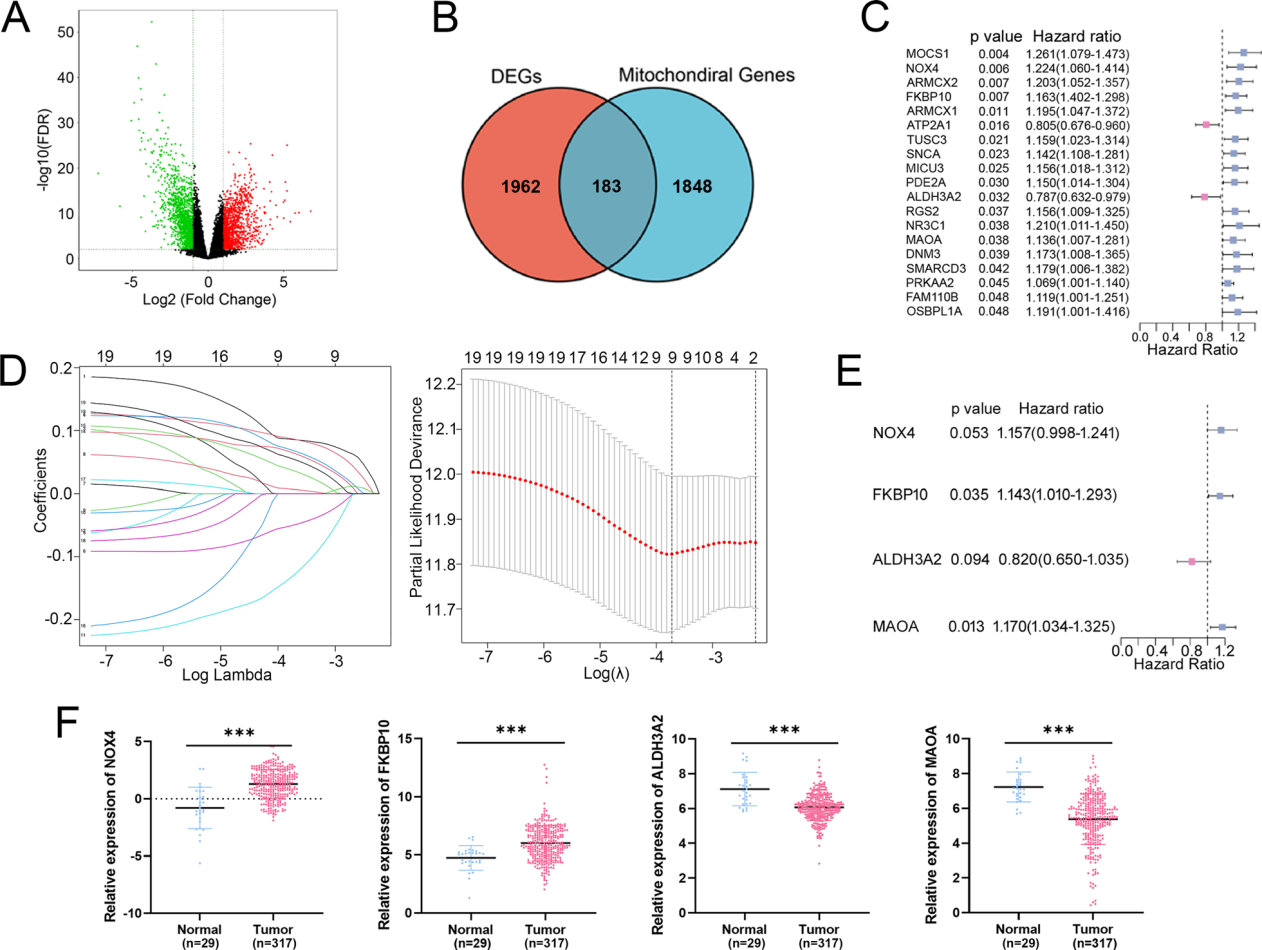
Identification of DEGs related to mitochondrion and construction of prognostic risk model using TCGA-STAD cohort. **A** Volcano plot of 2381 DEGs in STAD tumor and normal groups. **B** Venn diagram showed that the overlap of 2381 DEGs and 2030 mitochondrial genes led to 183 hub genes being identified. **C** Univariate Cox regression analysis revealed 19 genes were associated with prognosis of patients with STAD. **D** LASSO regression of the 19 OS-related genes. Cross-validation in the LASSO regression model to select the tuning parameter. The abscissa shows the log (λ) value, and the ordinate shows partial likelihood deviance. The red dots in the figure show partial likelihood deviations ± standard error for diverse tuning parameters. **E** Multivariable Cox regression analysis revealed 4 genes were associated with prognosis of patients with STAD. **F** Gene expressions of the 4 prognosis-related genes in TCGA-STAD. *P* values were showed as: ***P < 0.001

The general workflow of our current study was presented in Fig. 1. As shown in Additional file 10: Table S7, 2381 DEGs, including 2145 protein-coding genes, were screened and visualized via volcano maps between normal and tumor groups (Fig. 2A, Additional file 10: Table S8). Next, combined analysis for selected mitochondrial-related genes from the GSEA and 2145 DEGs from our study were performed to filter out 183 candidate mitochondrial-related DEGs in STAD (Fig. 2B, Additional file 10: Table S9).

GO enrichment analysis were then carried out to uncover important roles of mitochondrial-related DEGs in STAD. These DEGs potentially participated in small molecule catabolic process, regulation of mitochondrial organization, et al. Regarding the cellular component, they were mainly related to mitochondrial matrix, mitochondrial outer membrane, et al. In terms of molecular function, these DEGs were involved in tubulin binding, and ubiquitin-like protein ligase binding, et al. (Additional file 1: Fig. S1A, Additional file 10: Table S10). Moreover, KEGG pathway analysis was also applied to demonstrate important pathways being involved by these DEGs, such as lipid and atherosclerosis, Hepatitis B infection, Diabetic cardiomyopathy, and apoptosis, et al. (Additional file 1: Fig. S1B, Additional file 10: Table S11).

### Construction and validation of a mitochondrial-related risk signature

Based on above 183 mitochondrial-related DEGs, 19 genes were further selected as potential risk factors for the prognosis of patients with STAD through univariate Cox regression analysis (*P* < 0.05, Fig. 2C). The gene number was further narrowed down to 9 according to LASSO regression analysis and to 4 by multivariable Cox regression analysis (Fig. 2D, E). Finally, 4 mitochondrial-related DEGs, including NOX4, FKBP10, ALDH3A2 and MAOA, were utilized to establish a prognostic model for patients with STAD (Table 1).

**Table 1:** The information of 4 prognosis-related genes

Gene symbol	Gene ID	Full name	Location	Function of the encoded protein
NOX4	50507	NADPH oxidase 4	Membrane	The ROS generated by NOX4 have been implicated in numerous biological functions including signal transduction, cell differentiation and tumor cell growth
FKBP10	60681	FKBP prolyl isomerase 10	Mitochondria	FKBP10 localizes to the endoplasmic reticulum and acts as a molecular chaperone. Alternatively spliced variants encoding different isoforms have been reported, but their biological validity has not been determined
ALDH3A2	224	Aldehyde dehydrogenase 3 family member A2	Endoplasmic reticulum	Aldehyde dehydrogenase isozymes are thought to play a major role in the detoxification of aldehydes generated by alcohol metabolism and lipid peroxidation
MAOA	4128	Monoamine oxidase A	Mitochondria	This gene is one of two neighboring gene family members that encode mitochondrial enzymes which catalyze the oxidative deamination of amines, such as dopamine, norepinephrine, and serotonin

As shown in Fig. 2F, higher expressions of NOX4 and FKBP10, and lower expressions of ALDH3A2 and MAOA were observed in tumor samples compared with the normal tissues, respectively. Moreover, the immunohistochemistry results from HPA database showed that FKBP10 was upregulated in gastric cancer tissues, while ALDH3A2 and MAOA were downregulated in gastric cancer tissues, when compared with corresponding non-cancerous tissues (Additional file 1: Fig. S1C). Further K-M analysis demonstrated that patients with higher expression of NOX4 (*P* = 0.030), FKBP10 (*P* = 0.040), and MAOA (*P* = 0.018) had a shorter OS than those with lower expression, respectively (Additional file 1: Fig. S1D). However, the patients with higher level of ALDH3A2 had a better OS than those with lower expression, even though it is a bit beyond statistically significant difference (*P* = 0.052, Additional file 1: Fig. S1D).

Then, the risk score for each patient with STAD in both training and validation cohorts was computed based on the following formula:

$${\text{Risk score}}\, = \,0.{157}*{\text{MAOA}} - 0.{198}*{\text{ALDH3A2}}\, + \,0.{133}*{\text{FKBP1}}0\, + \,0.{146}*{\text{NOX4}}{.}$$

**Fig. 3:**
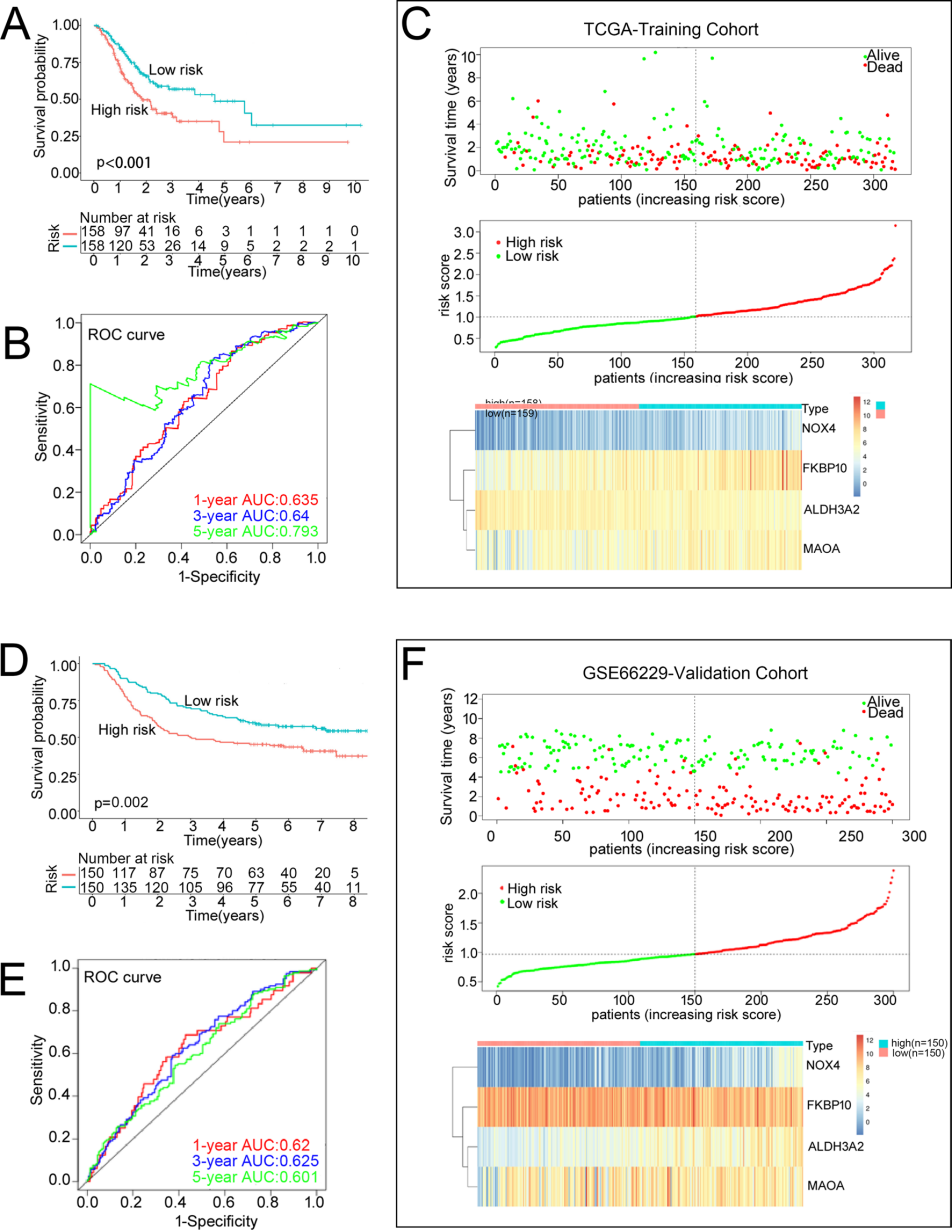
Assessing the performance of the prognostic risk model in the training and validation cohort. **A**, **D** Kaplan–Meier curves of the OS of patients in the high- and low-risk groups in the TCGA-STAD training cohort (**A**), and GSE66229 cohort (**D**). **B**, **E** ROC curves for predicting 1-, 3-, and 5-year OS in the TCGA-STAD training cohort (**B**), and GSE66229 cohort (**E**). **C**, **F** Distribution of risk score, survival status (red dots indicate dead, blue dots indicate alive) and the gene expression of 4 model genes in the TCGA-STAD training cohort (**C**), and GSE66229 cohort (**F**)

Patients were divided into high-risk and low-risk subgroups based on the median risk score. K–M curves showed that patients in high-risk group had worse OS (*P* = 0.0009, Fig. 3A). To assess the accuracy of prognostic risk models in predicting 1-, 3-, and 5-year OS, ROC curves were plotted with AUC values of 0.635, 0.640, and 0.793, respectively (Fig. 3B). The relationship between the risk score and the survival time, survival status, and risk ranking, and a heatmap of the expressions of the 4 genes were shown in Fig. 3C. Taken together, these results demonstrated the robustness of our risk model in predicting the prognosis of patients with STAD.

The robustness of the prognostic risk model was further validated in GSE66229 and GSE15459 datasets. In line with that of the training cohort (TCGA-STAD), patients in high-risk group also had worse prognosis in the validation cohorts (Fig. 3D, Additional file 2: Fig. S2A). In GSE66229 dataset, the AUC values of the ROC curve for 1-year, 3-year and 5-year survival were 0.620, 0.625, and 0.601, respectively (Fig. 3E). Corresponding AUC values of 0.620, 0.647, and 0.657 were observed in GSE15459 dataset (Additional file 2: Fig. S2B). The higher the risk score, the worse the survival (Fig. 3F, Additional file 2: Fig. S2C). The heatmaps of the expressions of the 4 genes were shown in Fig. 3F, Additional file 2: Fig. S2C. Consistent with the TCGA-STAD training cohort, the expressions of the NOX4 and FKBP10 were significantly up-regulated, while the expressions of the MAOA and ALDH3A2 were significantly down-regulated in STAD in GSE66229 validation cohort (Additional file 2: Fig. S2D). Next, we systematically analyzed the relationship between the risk score and clinical characteristics in STAD. The risk scores were remarkably higher in patients with H pylori infection, and cystic, mucinous and serous neoplasms (Additional file 3: Fig. S3). Nevertheless, no differences were observed in the mean of risk score among the groups of age, gender, T stage, N stage, M stage, tumor stage, family history of GC, grade, and reflux history (Additional file 3: Fig. S3). The clinical characteristics of the low-risk and high-risk subgroups were then compared, and the difference of Gender (*P* = 0.006), N stage (*P* = 0.043), H pylori infection (*P* = 0.035), disease type (*P* = 0.046) and survival status (*P* = 0.008) among the two risk subgroups reached statistical significance (Table 2).

**Table 2:** Clinical characteristics between low- and high-risk groups

Variables	Low risk No. (%)	High risk No. (%)	P value
Age (years)			0.466
< 67	73 (45.91%)	79 (50.00%)	
≥ 67	86 (54.09%)	79 (50.00%)	
Gender			**0.006**
Male	90 (56.60%)	113 (71.52%)	
Female	69 (43.40%)	45 (28.48%)	
T stage			0.385
T1/2	44 (27.67%)	37 (23.42%)	
T3/4	115 (72.33%)	121 (76.58%)	
N stage			**0.043**
N0/1	97 (61.01%)	84 (53.16%)	
N2/3	62 (38.99%)	69 (43.67%)	
NX	0 (0.00%)	5 (3.16%)	
M stage			0.624
M0	145 (91.19%)	141 (89.24%)	
M1	8 (5.03%)	12 (7.59%)	
MX	6 (3.77%)	5 (3.16%)	
Tumor stage			0.958
I/II	75 (47.17%)	75 (47.47%)	
III/IV	84 (52.83%)	83 (52.53%)	
Family history of stomach cancer			0.205
Yes	5 (3.14%)	10 (6.33%)	
No	122 (76.73%)	121 (76.58%)	
N/A	32 (20.13%)	27 (17.09%)	
*H.* *pylori* infection			**0.035**
Yes	6 (3.77%)	11 (6.96%)	
No	80 (50.31%)	49 (31.01%)	
N/A	73 (45.91%)	98 (62.03%)	
Grade			0.152
G1	3 (1.89%)	5 (3.16%)	
G2	62 (38.99%)	46 (29.11%)	
G3	92 (57.86%)	101 (63.92%)	
GX	2 (1.26%)	6 (3.80%)	
Reflux history			0.988
Yes	19 (11.95%)	16 (10.13%)	
No	86 (54.09%)	72 (45.57%)	
N/A	54 (33.96%)	70 (44.30%)	
Disease type			**0.046**
Adenomas and adenocarcinomas	150 (94.34%)	139 (87.97%)	
Cystic, mucinous and serous neoplasms	9 (5.66%)	19 (12.03%)	
Survival status			**0.008**
Alive	105 (66.04%)	81 (51.27%)	
Dead	54 (33.96%)	77 (48.73%)	

Bold indicates *P* value ≤ 0.05 was considered statistically significant

### Construction of nomogram

The nomogram integrated the risk score and all important clinical features, which can be used to quantitatively predict the prognosis of patients and provide a reference for clinical decision making. In our study, risk score (*P* = 0.0005) and age (P = 0.020) were finally identified as prognostic indicators by using univariate and multivariate Cox regression analysis to construct nomogram (Table 3). As a result, a predictive nomogram integrating risk score (a score of 100) and age (a score of 67.5) for prognosis was constructed (Additional file 4: Fig. S4A). ROC curves showed that the AUC values of the nomogram were 0.651, 0.664, and 0.749 for 1-, 3-, and 5-years OS, respectively (Additional file 4: Fig. S4B). The calibration curve showed that the actual survival probabilities at 1-, 3- and 5-year were almost in accordance with the survival probabilities predicted by the nomogram model (Additional file 4: Fig. S4C). The decision curves showed that the nomogram model was better than other factors in predicting the prognosis in STAD (Additional file 4: Fig. S4D).

**Table 3:** Univariate and multivariate Cox regression analysis of various prognostic parameters in STAD patients

Variables	Patient (N = 317)	Univariate analysis		Multivariate analysis	
		HR [95% CI]	P value	HR [95% CI]	P value
Age					
< 67	152	1		1	
≥ 67	165	2.093[1.412, 3.103]	** < 0.001**	1.514[1.068, 2.146]	**0.020**
Gender					
Male	203	1			
Female	114	1.254 [0.851, 1.849]	0.253		
T stage					
T1	13	1			
T2	68	1.642[0.347, 7.763]	0.532		
T3	151	1.861[0.338, 10.249]	0.475		
T4	85	1.611[0.283, 9.160]	0.591		
N stage					
N0	98	1			
N1	83	1.453[0.729, 2.895]	0.288		
N2	65	1.040[0.426, 2.537]	0.932		
N3	66	1.658[0.691, 3.977]	0.257		
NX	5	1.732[0.213, 14.072]	0.607		
M stage					
M0	286	1			
M1	20	1.095[0.441, 2.717]	0.845		
MX	11	1.125[0.381, 3.323]	0.832		
Tumor stage					
I	44	1			
II	106	0.988[0.364, 2.679]	0.981		
III	135	1.203[0.318, 4.550]	0.785		
IV	32	2.785[0.652, 11.898]	0.167		
Family history of stomach cancer					
Yes	15	1			
No	243	0.946[0.541, 1.655]	0.845		
N/A	59	0.833[0.316, 2.196]	0.712		
*H.* *pylori* infection					
Yes	17	1			
No	129	0.983[0.583, 1.657]	0.949		
N/A	171	0.576[0.214, 1.550]	0.275		
Grade					
G1	8	1			
G2	108	2.529[0.322,19.847]	0.377		
G3	193	3.226[0.418, 24.870]	0.261		
GX	8	4.065[0.378, 43.697]	0.247		
Reflux history					
Yes	35	1			
No	158	0.972[0.567, 1.665]	0.917		
N/A	124	0.561[0.231, 1.364]	0.202		
Disease type					
Adenomas and adenocarcinomas	289	1			
Cystic, mucinous and serous Neoplasms	28	0.615[0.301, 1.256]	0.182		
Risk					
High	158	1		1	
Low	159	0.525[0.354, 0.780]	**0.001**	0.538[0.379, 0.765]	** < 0.001**

Bold indicates *P* value ≤ 0.05 was considered statistically significant

### Functional enrichment analysis of the DEGs in high-risk and low-risk groups

**Fig. 4:**
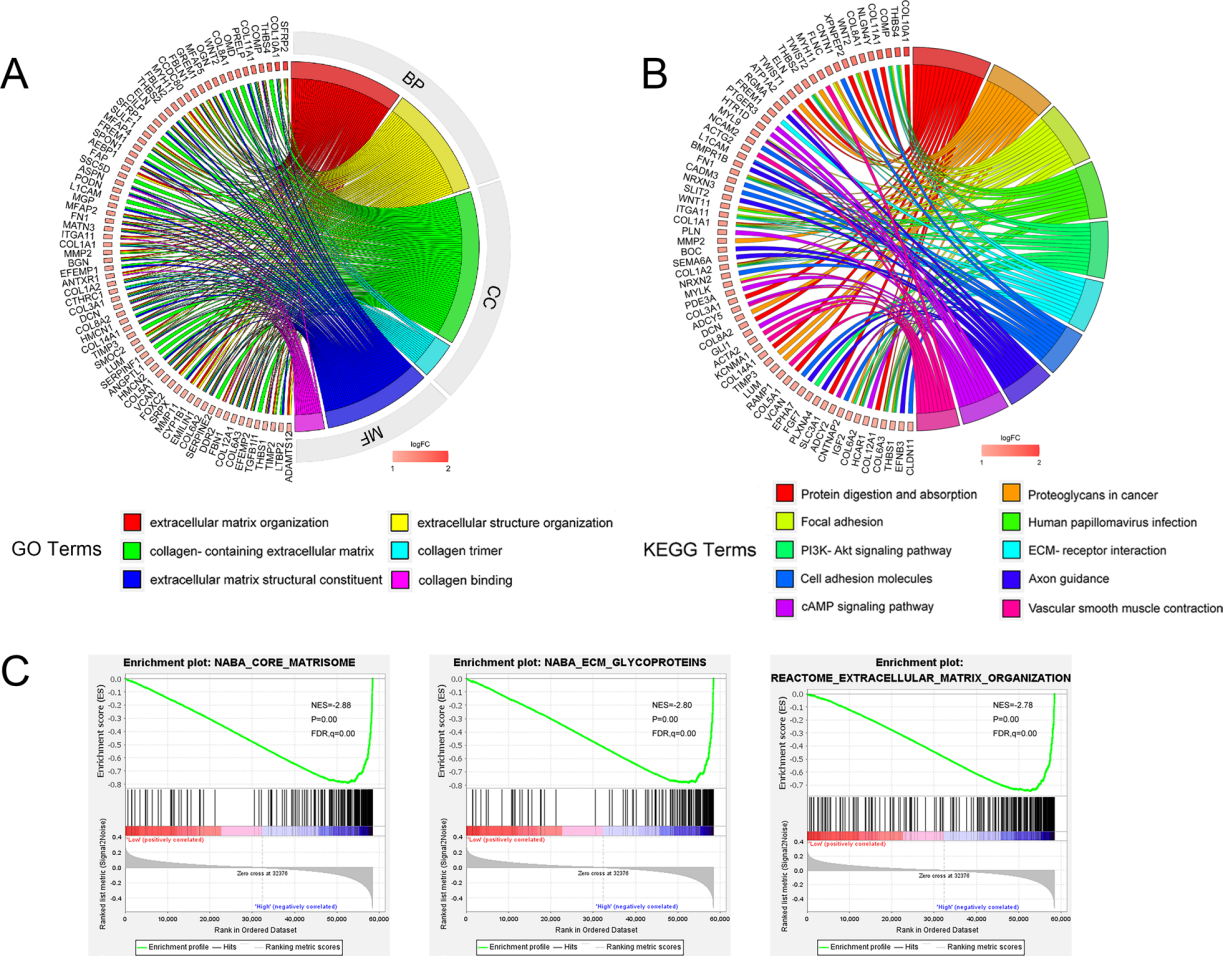
Enrichment analysis in high-risk group and the low-risk group. **A** Circle map. Bands with different colors in the right half circle symbolized 6 significant GO pathways, including biological process (BP), cellular component (CC), and molecular function (MF). The 6 pathways were enriched by genes listed in the left half circle. **B** Circle map. Bands with different colors in the right half circle symbolized top 10 significant KEGG pathways. The 10 top pathways were enriched by genes listed in the left half circle. **C** GSEA recognized different gene sets in the high-risk groups

We further conducted functional enrichment analyses of the 298 DEGs in high-risk and low-risk groups (Additional file 10: Table S12). GO enrichment analysis indicated that the differential genes annotated to biological processes were involved in extracellular matrix (ECM) organization and extracellular structure organization. Differential genes annotated to cellular component categories were mainly enriched in collagen-containing ECM and collagen trimer. Differential genes annotated to molecular function categories were mainly enriched in ECM structural constituent and collagen binding (Fig. 4A, Additional file 10: Table S13). The top 10 pathways obtained by KEGG analysis were: protein digestion and absorption, proteoglycans in cancer, focal adhesion, human papillomavirus infection, PI3K-Akt signaling pathway, ECM-receptor interaction, cell adhesion molecules, axon guidance, cAMP signaling pathway, and vascular smooth muscle contraction (Fig. 4B, Additional file 10: Table S14). GSEA results showed that risk score was significantly associated with ECM glycoproteins, core matrisome, ECM organization in high-risk group (Fig. 4C). The detailed GSEA results for the high-risk and low-risk groups were presented in Additional file 5: Fig. S5.

### Mitochondrial-related risk score was associated with TME signatures in STAD

**Fig. 5:**
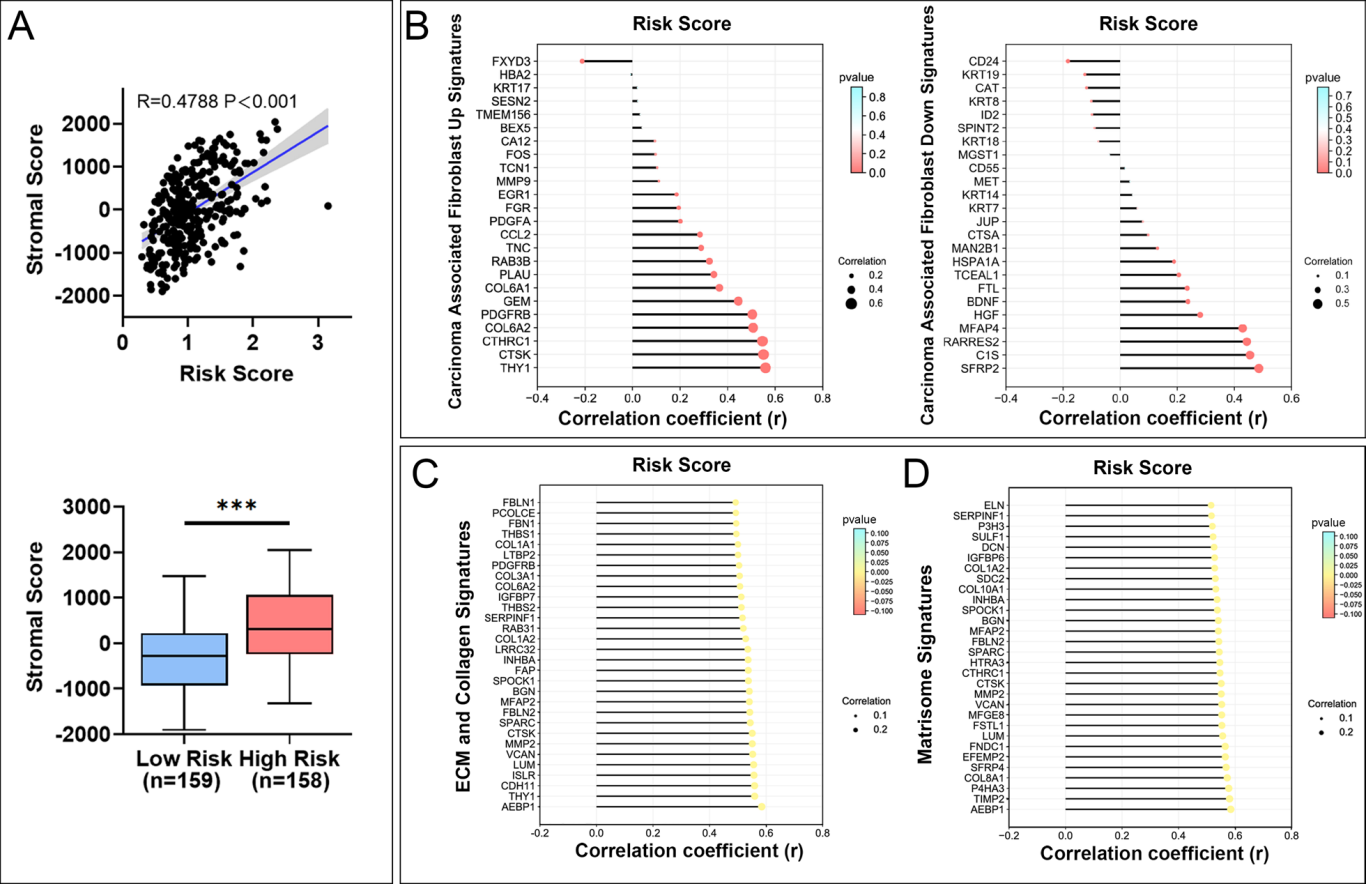
Risk score was associated with TME signatures in STAD. **A** Association between stromal score and risk score and its distribution in the low- and high-risk groups. **B** Correlation analysis for risk score and the expressions of carcinoma associated fibroblast (CAF) up and down signatures. **C** Correlation analysis for risk score and the expressions of ECM and collagen signatures. **D** Correlation analysis for risk score and the expressions of matrisome signatures. *P* values were showed as: ***P < 0.001

Given the TME-associated signal pathway was enriched through functional enrichment analyses, we explored the relationship between the risk score and the TME signatures. As shown in Fig. 5A, risk score was highly positively correlated with stromal score in STAD, and stromal score was higher in high-risk group compared to that in the low-risk group. We further investigated the relationship between risk score and TME components. Our results indicated that risk score had a significant and positive correlation with the expressions of the majority of carcinoma associated fibroblast (CAF) signatures (Fig. 5B), as well as ECM-collagen and matrisome signatures (Fig. 5C, D). Taken together, these results suggested a close relationship between mitochondrial-related risk score and TME signatures in STAD.

### Mitochondrial-related risk score was associated with immune signatures and immunotherapy responses in STAD

**Fig. 6:**
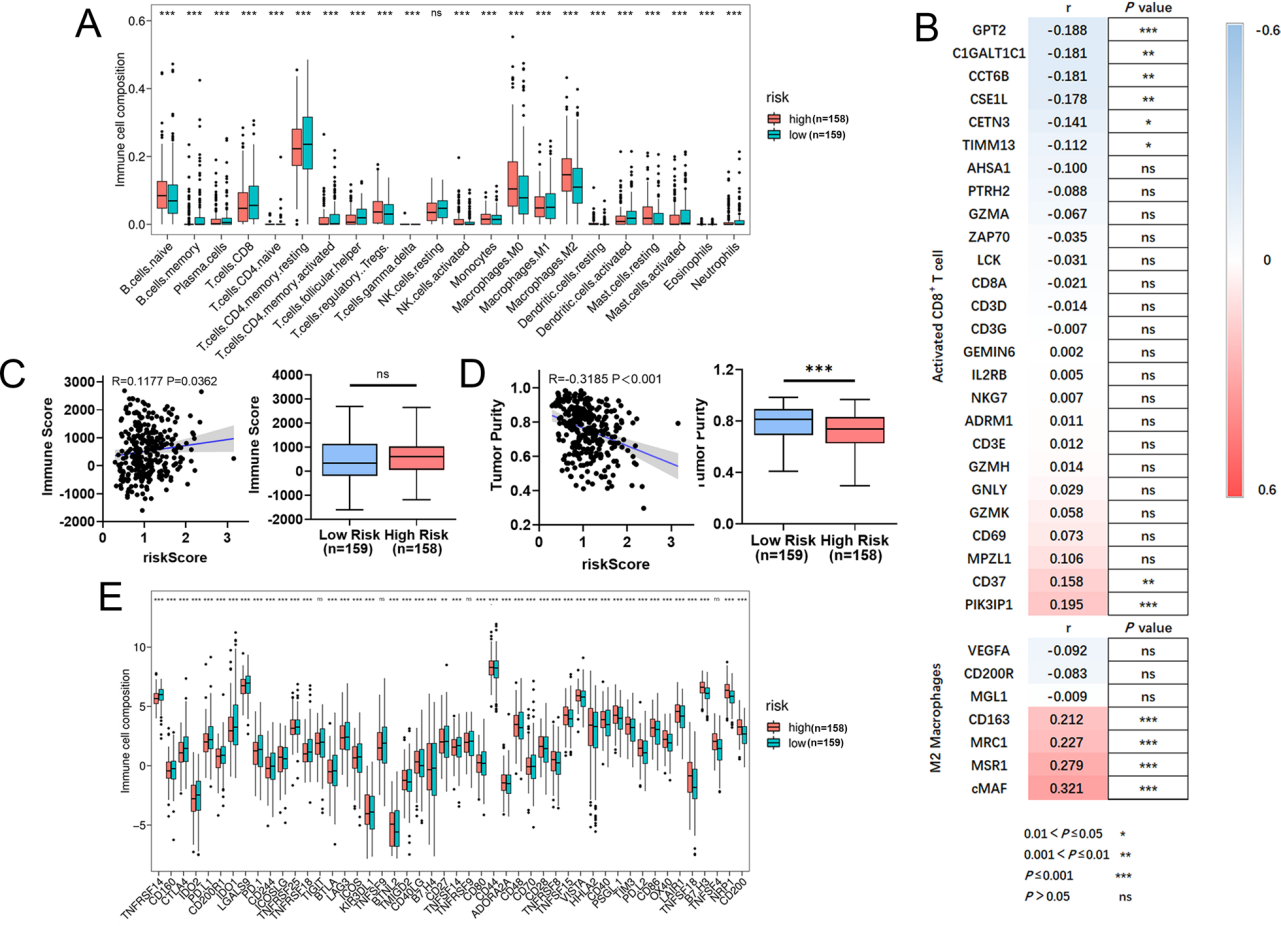
The different immune profiles between the low- and high-risk groups in the TCGA-STAD dataset. Two risk groups were divided based on the median risk score. **A** CIBERSORT analysis.** B** Correlation between risk score and the expressions of activated CD8^+^ T cell and M2 macrophages signatures. **C**, **D** ESTIMATE algorithm. **E** Expression variation of immune checkpoint. *P* values were showed as: ns not significant; *P < 0.05; **P < 0.01; ***P < 0.001

The tumor immune microenvironment was closely related with the therapeutic effects and prognosis of patients with malignant tumor. It is reasonable to check the relationship between the risk score and the immune cell infiltration in STAD. The contents of naive B cells, regulatory T cell (Tregs), M0 macrophage, and M2 macrophage were remarkably higher in the high-risk group. In contrast, CD8^+^ T cells and resting CD4^+^ T cells were higher in low-risk group (Fig. 6A, Additional file 6: Fig. S6A, B). Consistent with the above results, risk score was positively correlated with the expressions of the majority signatures of M2 macrophage, while negatively correlated with the expressions of the majority signatures of activated CD8^+^ T cell (Fig. 6B). The correlations between the risk score and the signatures of other immune cells were presented in Additional file 8: Fig. S8. Consistent with the previous studies [28], the ESTIMATE results showed that the patients in high-risk had higher immune score, and significantly lower tumor purity (*P* = 0.370), than those in the low-risk groups (Fig. 6C, D). Due to the positive correlation between the risk score, and matrisome and CAF signatures, as well as the negative correlation between the risk score and activated CD8^+^ T cells signatures, we speculated activated CD8^+^ T cells signatures was negatively correlated with the matrisome and CAF signatures. Interestingly, our results showed that the expression of activated CD8^+^ T cell signatures were negatively correlated with both matrisome and CAF signatures (Additional file 6: Fig. S6C). Taken together, these results strongly suggested the tumor immunosuppressive microenvironment might contributed to the worse prognosis of the patients with STAD in high-risk group, which needs to be validated in further study.

Nowadays, immune checkpoint inhibitors were studied and well applied in cancer immunotherapy. In the present study. Our results indicated that 43 immune checkpoints were considerably modulated in high-risk group (Fig. 6E). In addition, risk score was significantly positively correlated with the expression level of 7 immune checkpoints, including CD200, NRP1, TNFSF4, B7-H3, TNFSF18, LAIR1 and OX40 (*r* > 0.2, Additional file 6: Fig. S6D). Currently, the inhibitors for PD-1 and CTLA-4 are research hotspots in the treatment of advanced STAD. As shown in Fig. 6C, the expressions of PD-1, PD-L1 and CTLA-4 were significantly down-regulated in the high-risk group. Consistently, risk score was significantly negatively correlated with the expressions of PD1, PD-L1 and CTLA-4, respectively (Additional file 6: Fig. S6D).

**Fig. 7:**
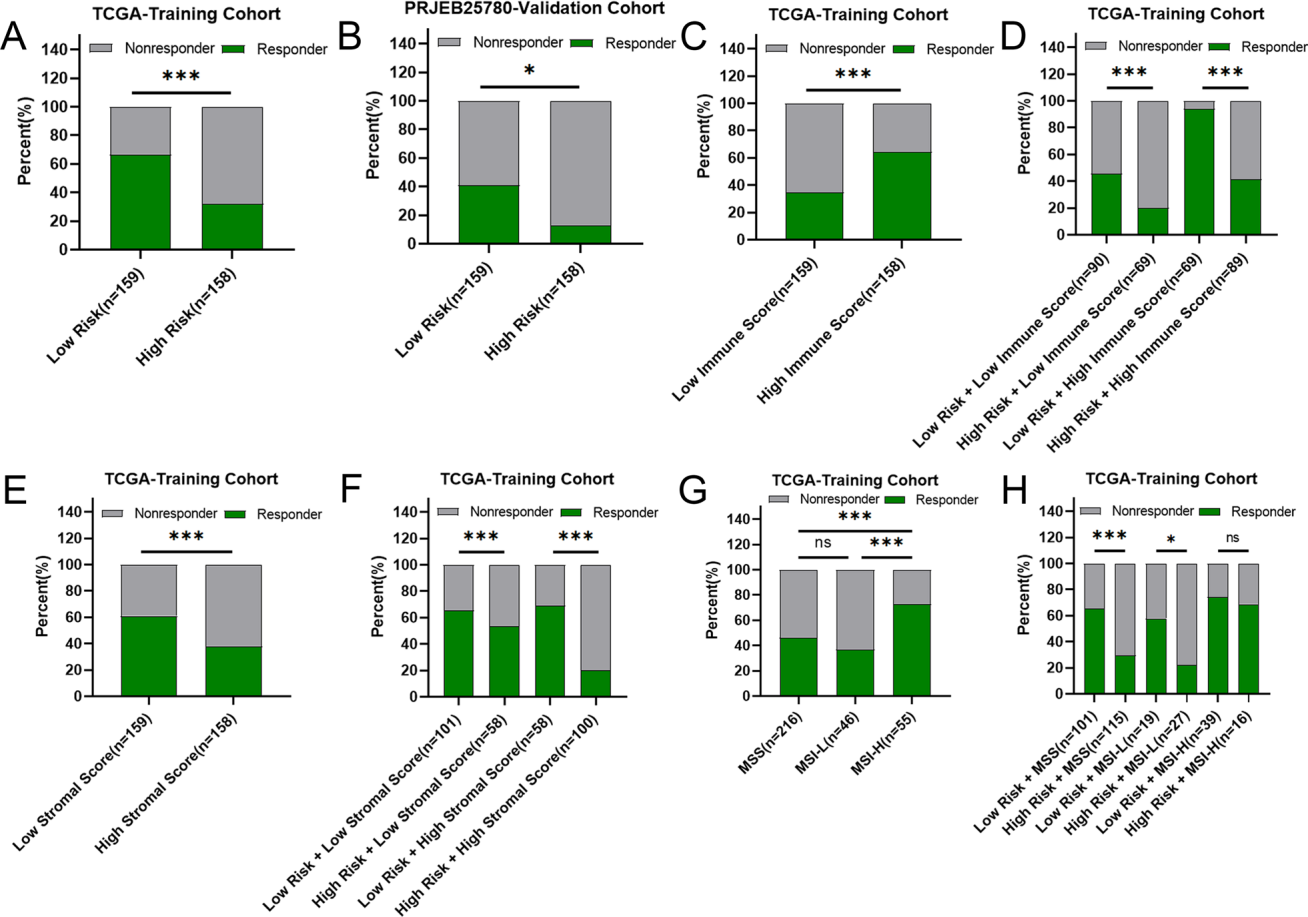
Risk score is a potential biomarker to predict benefits from immune therapies in STAD. **A** TIDE predicted the proportion of patients with response to immunotherapy in low-risk and high-risk groups. **B** The proportion of patients with response to immunotherapy in low-risk and high-risk groups in the PRJEB25780 immunotherapy cohort (45 patients with advanced gastric cancer who had received PD-L1 inhibitor treatment). **C** TIDE predicted the proportion of patients with response to immunotherapy in low-immune score and high-immune score groups. **D** TIDE predicted the proportion of patients of four groups based on the risk score and immune score with response to immunotherapy. **E** TIDE predicted the proportion of patients with response to immunotherapy in low-stromal score and high-stromal score groups. **F** TIDE predicted the proportion of patients of four groups based on the risk score and immune score with response to immunotherapy. **G** TIDE predicted the proportion of patients with response to immunotherapy in MSS, MSI-L and MSI-H groups. **H** TIDE predicted the proportion of patients of six groups based on the risk score and microsatellite status with response to immunotherapy. MSS, Microsatellite stability; MSI-L, Microsatellite Instability-Low; MSI-H, Microsatellite Instability-High. *P* values were showed as: ns not significant; ***P < 0.001

Given the above results, we further used the TIDE algorithm to evaluate the ability of risk score in predicting the responses to immunotherapy in STAD. Our results showed that risk score had a significantly positive correlation with TIDE score (Additional file 7: Fig. S7), indicating that patients in low-risk group received better response to immunotherapy. The immunotherapy response rate in high-risk group (32.28%) was significantly lower than that in low-risk group (66.67%) (Fig. 7A).

Next, PRJEB25780 cohort (PD-L1 inhibitor treatment for 45 patients with advanced gastric cancer [29]) was applied to validate whether the mitochondrial-related risk signature could accurately predict the responses to immunotherapy for patients with STAD. Consistent with the prediction results by TIDE, the immunotherapy respond rate in high-risk group (13.04%) was significantly lower than that in low-risk group (40.91%) in PRJEB25780 validation cohort (Fig. 7B).

As shown in Fig. 7C, the immunotherapy response rate in the low-immune subgroup (34.59%) was remarkably lower than that in the high-immune subgroup (64.56%). Interestingly, the immunotherapy response rate in low-risk group (45.56%) was remarkably higher than that in high-risk group (20.29%) in the subgroup with low-immune score. Moreover, the immunotherapy response rate in low-risk group (94.20%) was significantly higher than that in high-risk group (41.57%) in the subgroup with high-immune score, strongly suggesting that combined risk score and immune score was a robust indicator to predict the responses to immunotherapy in STAD (Fig. 7D). In addition, the immunotherapy response rate in the high stromal group (37.97%) was significantly lower than that in the low stromal group (61.01%) (Fig. 7E). In the low stromal subgroup, the immunotherapy response rates were 65.35% and 53.45% in low risk and high risk subgroup, respectively, which were similar to the low-stromal group (61.01%), indicating combined risk score and stromal score was not better than stromal score alone in predicting the response to immunotherapy in STAD patients with low-stromal score. However, the immunotherapy response rate in the low-risk + high-stromal group (68.97%) was significantly higher than that in high-stromal group (37.97%), whereas the immunotherapy response rate in the high-risk + high-stromal group (20.00%) was significantly lower than that in the high-stromal group (37.97%), strongly suggesting that combined risk score and stromal score can more accurately predict response to immunotherapy in STAD patients with high-stromal score (Fig. 7F).

The phenotype for microsatellite instability–high (MSI-H) is a distinct tumor subclass that is highly susceptible to immunotherapy. Consistent with the previous studies [30], the immunotherapy response rate MSI-H subgroup (72.73%) was remarkably higher than that in the MSS subgroup (46.30%) and MSI-L subgroup (36.96%) (all *P* = 0.0005, Fig. 7G). Furthermore, our results also showed that the immunotherapy response rate in low-risk group (65.35%) was remarkably higher than that in high-risk group (29.57%) in the subgroup with MSS. The immunotherapy response rate in low-risk group (57.89%) was also significantly higher than that in high-risk group (22.22%) in the subgroup with MSI-L. The above results strongly suggested that combination of risk-score and MSS/MSI-L can be used as a robust indicator to predict the response to immunotherapy in STAD (Fig. 7H).

### Mutation status of STAD patients in high-risk and low-risk groups

**Fig. 8:**
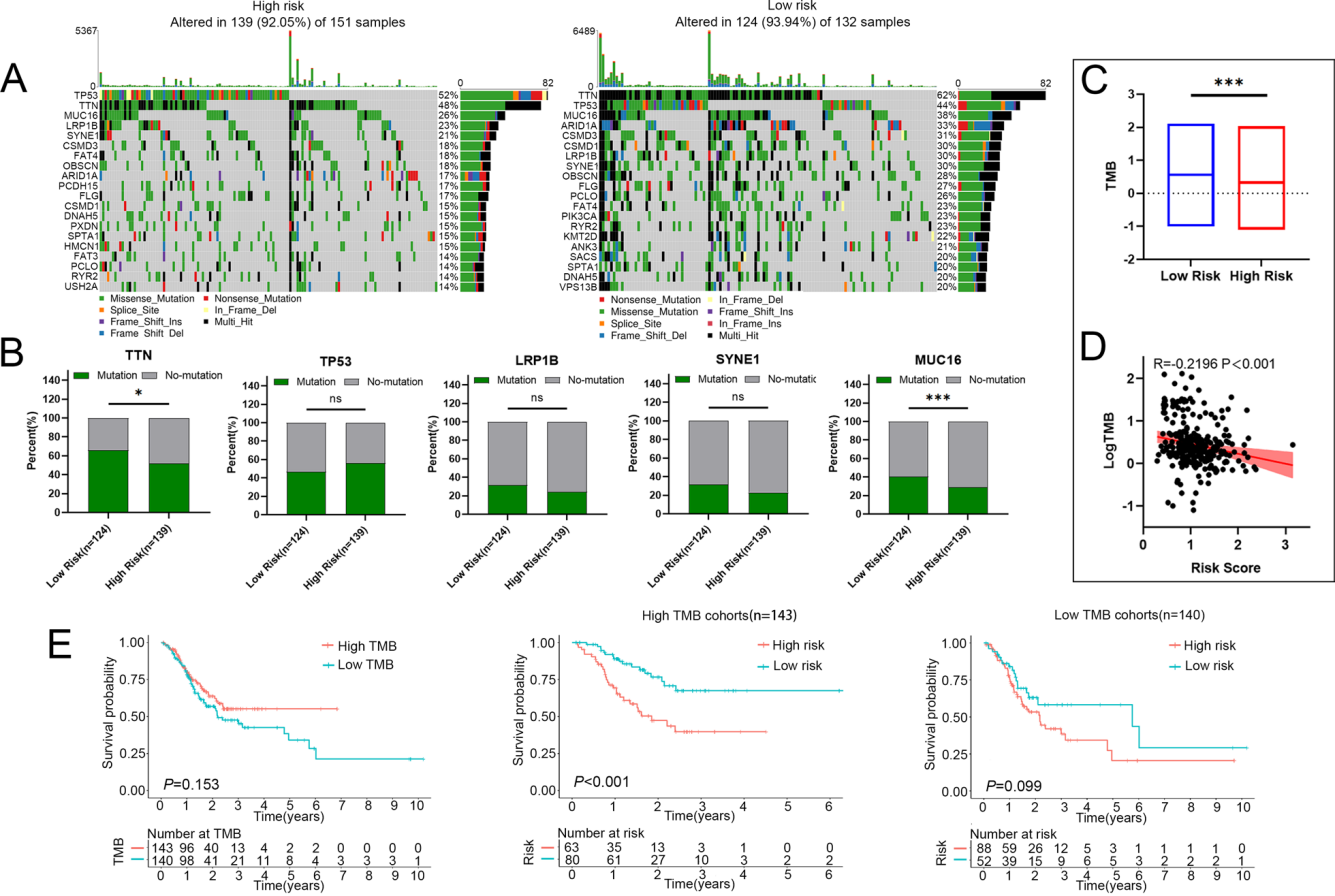
Mutation status in the high- and the low-risk groups in STAD. **A** The top 20 genes according to mutation frequency in low and high-risk groups, respectively. **B** Mutation rate of the top five mutant genes in high-risk and low-risk groups. **C** Relationship between the risk score and TMB. **D** Correlation between risk score and TMB score in STAD. **E** Kaplan–Meier curves of the OS of patients in the high- and low-TMB groups in the TCGA-STAD training cohort. *P* values were showed as: ns not significant; *P < 0.05; ***P < 0.001

Progressive accumulation of mutations throughout life can lead to cancer. Genome sequencing has revolutionized our understanding of somatic mutation in cancer, providing a detailed view of the mutational processes and genes that drive cancer [31]. Therefore, we mapped the mutation landscape of STAD in both the high risk and low risk groups, and analyzed the relationship between risk score and mutation profile. As shown in Fig. 8A, the top 20 high-frequency mutated genes in high-risk and low-risk group were presented. TP53, TTN, MUC16, LRP1B, SYNE1, CSMD3, FAT4, OBSCN, ARID1A, FLG, CSMD1, DNAH5, SPTA1, PCLO and RYR2 were the common high-frequency mutation genes in both groups. In top 5 mutant genes, the mutation rates of TTN and MUC16 were significantly decreased in high-risk group (Fig. 8B).

Besides, accumulating evidences supported that TMB functioned as a potentially predictive biomarker for multiple applications, including the biomarker for response to immunotherapy in malignancies [32–36]. Our data showed that TMB in high-risk group was significantly lower than that in low-risk group, and the risk score had a significant negative correlation with TMB in STAD (Fig. 8C, D). Interestingly, the higher TMB tended to have a better OS compared with the lower TMB, but without a statistically significant difference (*P* = 0.153, Fig. 8E). Moreover, low-risk group tended to have a better OS compared with high-risk group in high TMB subgroup. The similar results were obtained in the low TMB subgroup, but without a statistically significant difference (*P* = 0.099, Fig. 8E). Taken together, these results strongly suggested that combination of risk score and TMB might be a valuable biomarker for predicting the prognosis for STAD patients (Fig. 8E).

### Risk score predicts therapeutic benefits in STAD

**Fig. 9:**
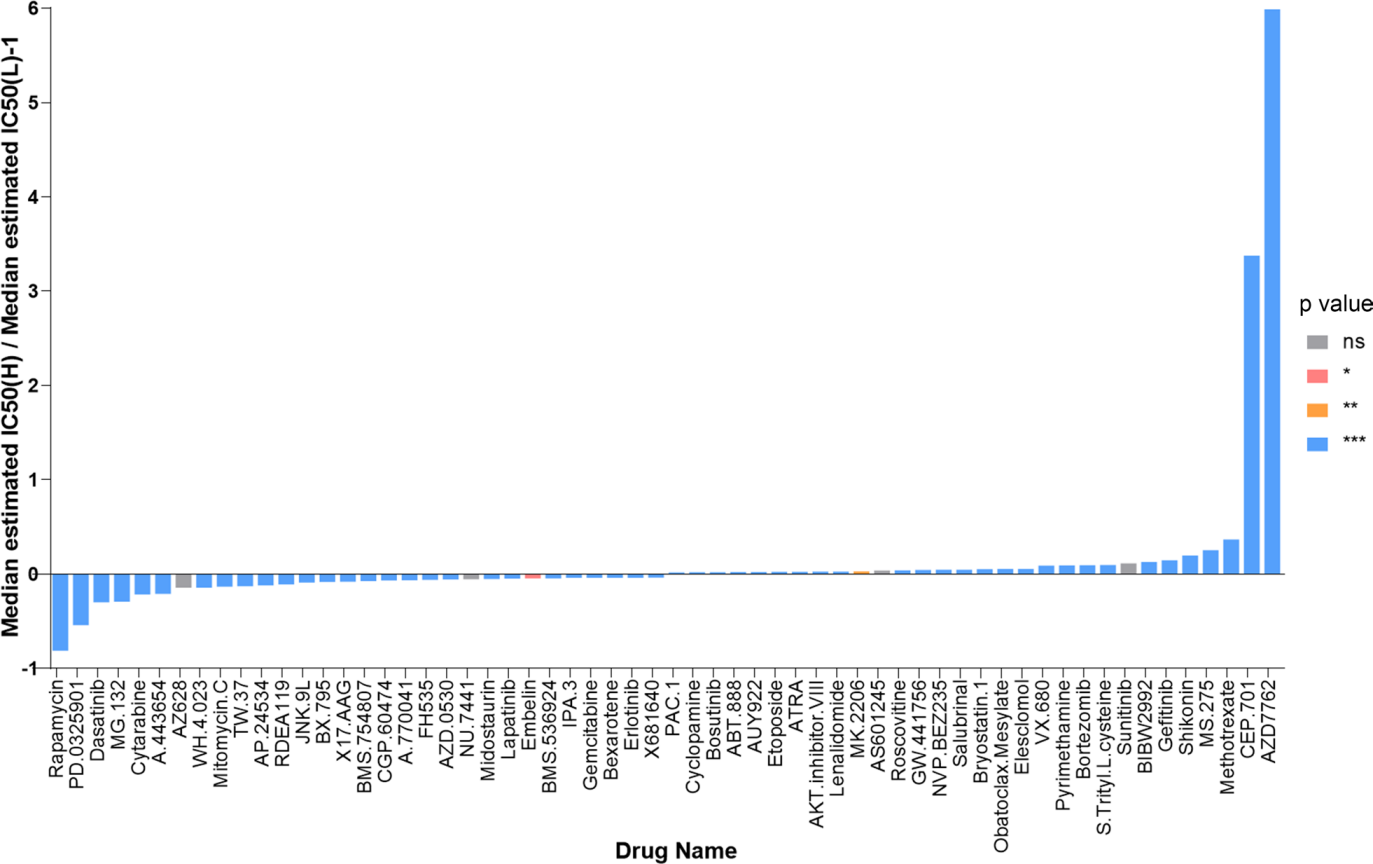
Risk score predicts drug therapeutic benefits in STAD. Proportion of normalized IC50 value of the 60 drugs between the low-risk and high-risk groups. P values were showed as: ns not significant; *P < 0.05; **P < 0.01; ***P < 0.001

To find the potency of risk score as an index for predicting the response to drugs (including chemotherapy, targeted therapy, and immunotherapy) in STAD, we inferred the IC50 value of the 138 drugs in TCGA-STAD patients. We found that patients in high-risk group might be more sensitive to rapamycin, PD-0325901, dasatinib, et al., whereas patients in low-risk group might be more sensitive to AZD7762, CEP-701, methotrexate, et al., which could provide a reliable reference for clinical treatment (Fig. 9). Detailed information on the top 10 sensitive drugs for the high-risk and low-risk subgroup was illustrated in Tables 4 and 5.

**Table 4:** Detailed information of the top 10 sensitivity drugs in high-risk groups

Drug name	Introduction	Drug target	Drug target pathway
Rapamycin	Drugs that selectively target mTORC1 are expected to impair cancer metabolism and are considered promising anti-cancer therapies	MTORC1	PI3K/MTOR signaling
PD.0325901	Mirdametinib (PD-0325901) is an oral, non-ATP-competitive, highly selective, and potent small-molecule inhibitor of MEK1 and MEK2	MEK1, MEK2	ERK MAPK signaling
Dasatinib	Dasatinib is an orally available short-acting dual ABL/SRC tyrosine kinase inhibitor (TKI). It potently inhibits BCR-ABL and SRC family kinases (SRC, LCK, YES, FYN), but also c-KIT, PDGFR-a and PDGFR-b, and ephrin receptor kinase	ABL, SRC, Ephrins, PDGFR, KIT	RTK signaling, kinases
MG-132	The peptide-aldehyde proteasome inhibitor MG132 (carbobenzoxyl-l-leucyl-l-leucyl-l-leucine) induces the apoptosis of cells by a different intermediary pathway. Although the pathway of induction of apoptosis is different, it plays a crucial role in anti-tumor treatment	Proteasome, CAPN1	Protein stability and degradation
Cytarabine	Cytarabine (molecular formula: C9H13N3O5) interferes with DNA synthesis, acting on DNA/RNA polymerase (and other nucleotide reductase enzymes), reducing cell ability to replicate	Antimetabolite	Other
A-443654	A-443654, a specific Akt inhibitor, interferes with mitotic progression and bipolar spindle formation. A-443654 induces G2/M accumulation, defects in centrosome separation, and formation of either monopolar arrays or disorganized spindles	AKT1, AKT2, AKT3	PI3K/MTOR signaling
AZ628	AZ628 is a hydrophobic Raf-kinase inhibitor currently in clinical trial of various cancer	BRAF	ERK MAPK signaling
WH-4–023	WH-4-023 is a LCK inhibitors	SRC, LCK	Other, kinases
Mitomycin.C	Mitomycin C (MMC) is an alkylating agent with extraordinary ability to crosslink DNA, preventing DNA synthesis	DNA crosslinker	DNA replication
TW.37	TW-37 is a novel, potent and non-peptide Bcl-2 small-molecule inhibitor	BCL2, BCL-XL, MCL1	Apoptosis regulation

**Table 5:** Detailed information of the top 10 sensitivity drugs in low-risk groups

Drug name	Introduction	Drug target	Drug target pathway
AZD7762	AZD7762 is a checkpoint kinase 1 (Chk 1) inhibitor, which has been reported to sensitize many tumor cells to DNA damage	CHEK1, CHEK2	Cell cycle
CEP-701	CEP‐701 is an inhibitor of tyrosine kinase. Treatment with CCEP-701 modulates various signalling pathways and leads to cell growth arrest and programmed cell death in several tumour types	FLT3, JAK2, NTRK1, NTRK2, NTRK3	Other, kinases
Methotrexate	Methotrexate (MTX) is a commonly used antimetabolite, which inhibits folate and DNA synthesis to be effective in the treatment of various malignancies	Antimetabolite	DNA replication
MS-275	MS-275, a selective class I inhibitor of histone deacetylase (HDAC), exerts anti-tumor activity in various cancer types, including multiple myeloma (MM)	HDAC1, HDAC3	Chromatin histone acetylation
Shikonin	Many studies have demonstrated that shikonin exerts strong anticancer effects on various types of cancer by inhibiting cell proliferation and migration, inducing apoptosis, autophagy, and necroptosis	Not defined	Other
Gefitinib	Gefitinib is an orally active, selective epidermal growth factor receptor-tyrosine kinase inhibitor	EGFR	EGFR signaling
BIBW2992	BIBW2992 is an irreversible blocker of the ErbB family, acting at the tyrosine kinases of these proteins. Further investigations for the treatment of many other tumors with BIBW2992, e.g., HNSCC and breast cancer, are ongoing	ERBB2, EGFR	EGFR signaling
Sunitinib	Sunitinib is a tyrosine kinase inhibitor indicated for the treatment of gastrointestinal stromal tumor, advanced renal cell carcinoma, and pancreatic neuroendocrine tumors	PDGFR, KIT, VEGFR, FLT3, RET, CSF1R	RTK signaling
S-Trityl-L-cysteine	S-Trityl-L-cysteine (STLC) is a well-recognized lead compound known for its anticancer activity owing to its potent inhibitory effect on human mitotic kinesin Eg5	KIF11	Mitosis
Bortezomib	Bortezomib (BTZ) is the first proteasome inhibitor approved by the Food and Drug Administration. It can bind to the amino acid residues of the 26S proteasome, thereby causing the death of tumor cells	Proteasome	Protein stability and degradation

### Experimental verification of hub gene expression in STAD

**Fig. 10:**
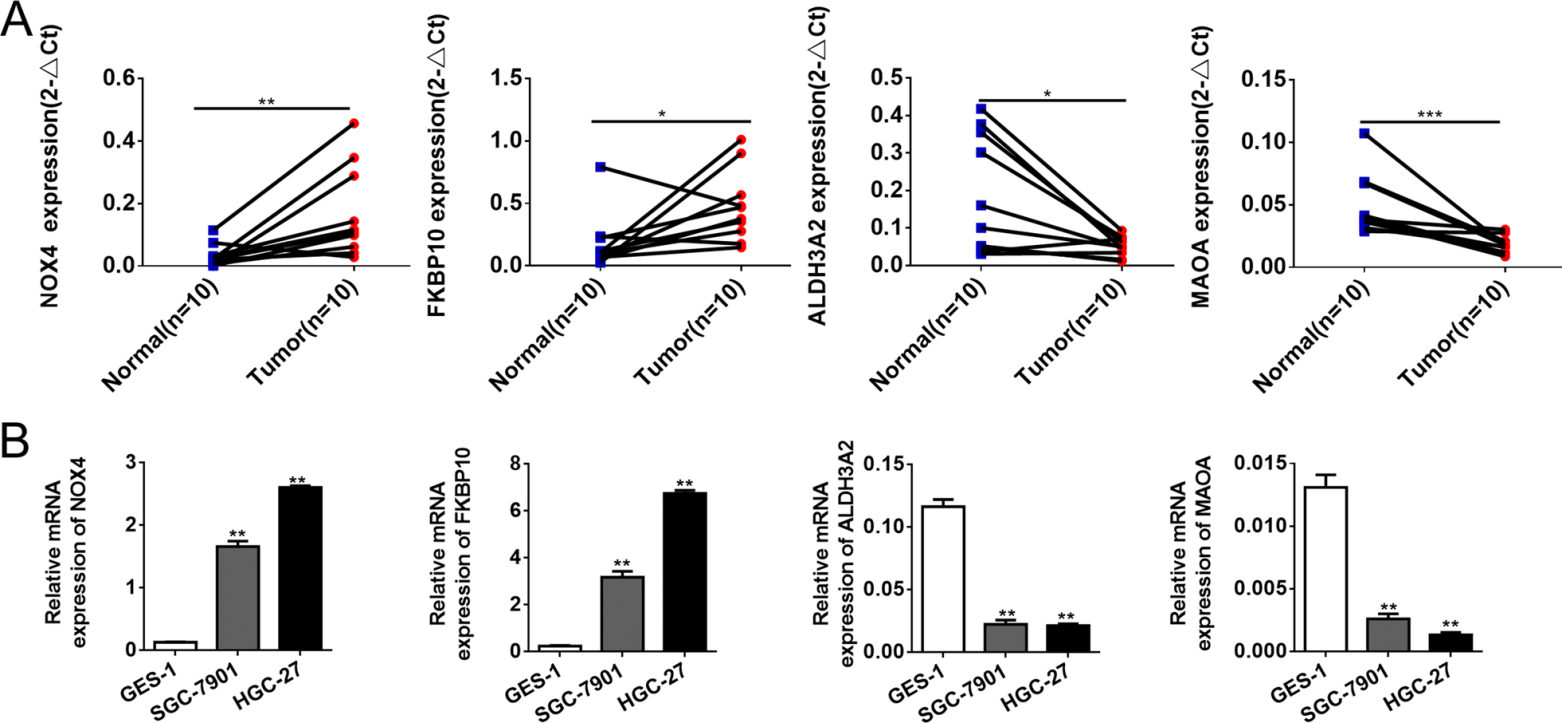
Experimental verification of 4 genes expression in STAD. **A** Expression of 4 genes in 10 paired STAD tissues and normal tissues was evaluated by qRT-PCR. **B** Expression of 4 genes in a human normal gastric epithelial cell line GSE-1 and human gastric cancer cell lines through qRT-PCR

**Fig. 11:**
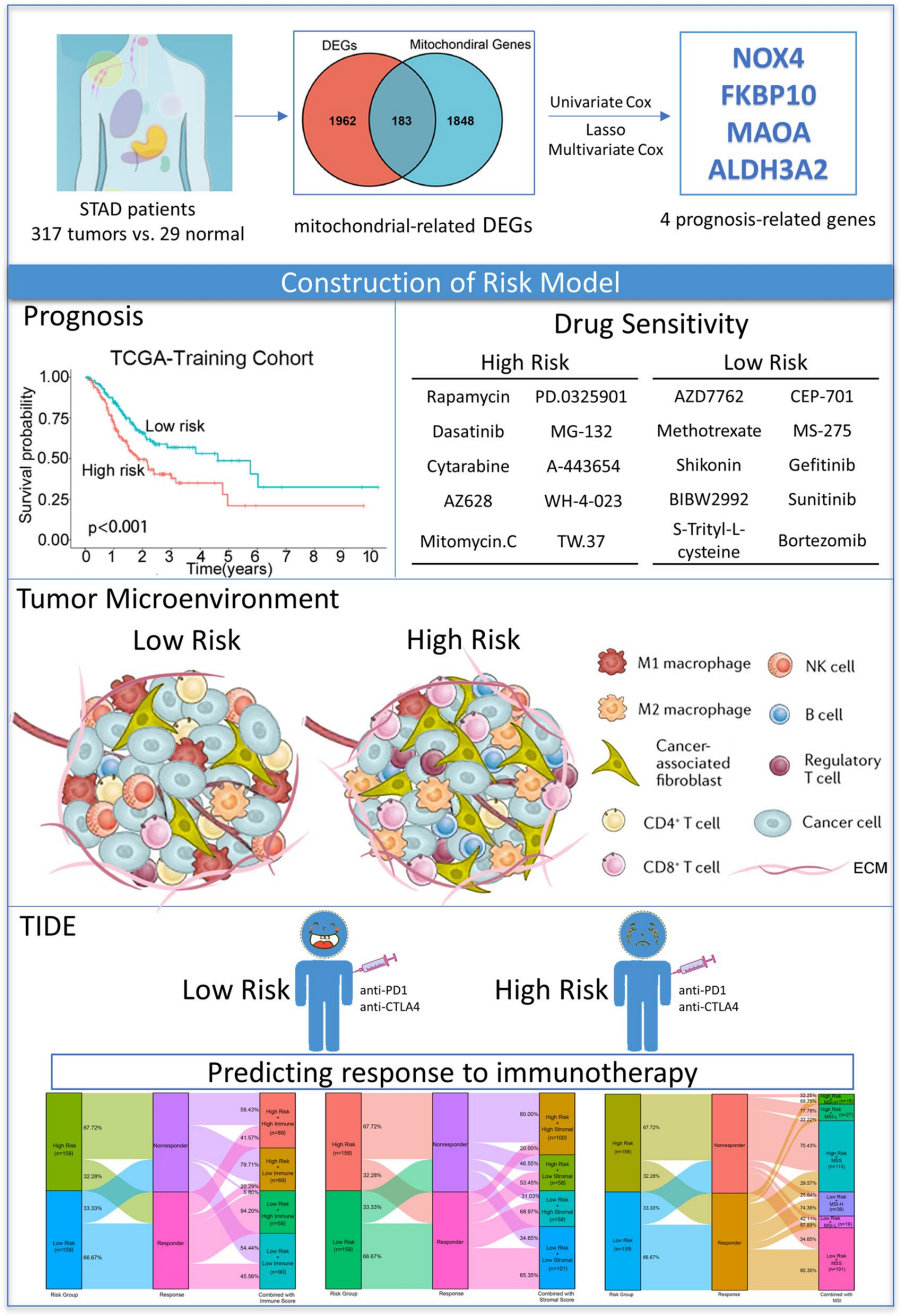
Graph summarization. The work summary graph of this study

To verify the expressions of hub genes in STAD samples, qRT-PCR was conducted on 10 pairs of STAD tumor and paired adjacent normal tissues. As shown in Fig. 10A, the expressions of NOX4 and FKBP10 were significantly higher in STAD tumor tissues than those in the paired adjacent normal tissues, respectively (all *P* < 0.05). The expressions of ALDH3A2 and MAOA were significantly lower in STAD tumor tissues than those in the paired adjacent normal tissues, respectively (all *P* < 0.05, Fig. 10A). We also verified the expressions of the four hub genes in a human normal gastric epithelial cell line GES-1 and human gastric cancer cell lines through qRT-PCR. Our results showed that the expressions of NOX4 and FKBP10 were significantly higher than in the normal gastric epithelial cell line GSE-1 (all *P* < 0.05, Fig. 10B), while the expressions of ALDH3A2 and MAOA were significantly lower than in the normal gastric epithelial cell line GSE-1 (all *P* < 0.05, Fig. 10B). These results supported our hypothesis and provide solid evidence for the rationality of choosing these four genes for prognostic model construction (see Fig. 11).

## Discussion

GC remains a frequent cancer worldwide with high incidence and mortality globally [1]. Effective biomarkers are still missing even though 3 biomarkers (HER2, MSI-H and PD-L1) have been proven to predict the responses of targeted therapy in GC [37]. Therefore, identifying more effective biomarkers for targeted therapy and prognosis prediction is highly demand. Mitochondria were important pharmacological targets due to their critical roles in cell proliferation and death. The mitochondrial energy metabolisms are now known to be reprogrammed to meet the challenges of high energy demand, with better use of available fuels for malignant cell growth and migration [38]. Thus, mitochondria play a vital and multifunctional role in tumor occurrence and development, and targeting mitochondria provides therapeutic opportunities [39]. A growing body of research showed that mitochondrial-related genes can be used as biomarkers for the diagnosis and treatment of malignancies.

NOX4 has been identified as a biomarker and therapeutic target for a variety of human cancers. NOX4 was upregulated in pancreatic cancer and was involved in the development of pancreatic cancer by promoting cell proliferation, regulating cell metabolism, and mediating angiogenesis, suggesting NOX4 was a potential therapeutic target for pancreatic cancer [40]. Up-regulation of NOX4 predicted worse prognosis and accelerated tumor growth in colorectal carcinoma [41]. Moreover, it has been shown that NOX4 recruited M2-macrophages via ROS/PI3K signaling pathway-dependent cytokines production, thus contributing to the cell division in NSCLC [42]. Consistent with the previous studies, NOX4 in our model was significantly up-regulated in STAD, and high level of NOX4 was associated with worse prognosis in patients with STAD.

It has been reported that a cancer-specific molecular mechanism for NSCLC was related with FKBP10-dependent protein translation. The expression of FKBP10 was positive in cancer lesions [43]. Li et al. reported that FKBP10 silencing decreased the expression of integrin αV and integrin α6, and P-AKT, suggesting that FKBP10 might promote metastasis [44]. FKBP10 was up-regulated in GC tissues and might be a reliable therapeutic target in GC [45]. Consistent with the above studies, our results showed that the high expression of FKBP10 was related with high risk, and predicated poor prognosis of STAD patients. Consistent with the previous studies, FKBP10 in our model was significantly up-regulated in STAD, and high level of FKBP10 was associated with worse prognosis in patients with STAD.

It has been shown that ALDH3A2 was overexpressed in low-grade GC compared with high-grade GC, and patients with low expression of ALDH3A2 had worse OS than those with high ALDH3A2 expression. ALDH3A2 was reported as a reliable biomarker for the immunotherapy, as well as an independent predictor for the prognosis of GC [46]. In renal clear cell carcinoma, low level of ALDH3A2 was related with shorter survival [47]. Consistently, ALDH3A2 was significantly down-regulated in STAD. However, ALDH3A2 couldn’t effectively predict the prognosis for patients with STAD, and this inconsistent effects of ALDH3A2 on prognosis may be caused by the different inclusion and exclusion criteria across different study, which needs more comprehensive investigation.

MAOA exerted different biological effects in different tumors. MAOA was found to be involved in mitochondrial dysfunction, and promoted malignant growth and metastasis in gastric cancer [48]. MAOA promoted prostate cancer progression by increasing cell growth and cancer stem cells, which suggested that MAOA might be a potential therapeutic target for the treatment of prostate cancer [49]. In the present study, MAOA was significantly down-regulated in STAD. However, the high level of MAOA was associated with worse prognosis in patients with STAD, which need to be validated in more samples in further study.

Currently, many biomarkers were applied for prognostic prediction of GC, such as NOX4, FKBP10 and ALDH3A2, but most of them are studied for a single biomarker [44–46, 50]. Increasing evidences indicated that prognostic model constructed by multi-genes as a prognostic index was more comprehensive and effective than single gene in kinds of malignancies. For instance, Nie et al. constructed a GC prognosis model based on metabolic signature, which were mainly related to the dysregulation of the metabolic microenvironment [51]. Wu et al. constructed a immune-related prognostic model [52]. As the dysfunction and dysregulation of mitochondria have been associated with cancer, we constructed a STAD prognostic model based on mitochondrial-related genes, which could effectively predict the prognosis for patients with STAD.

The DEGs between the high-risk and low-risk groups were mainly enriched to the extracellular matrix (ECM) and focal adhesion pathway. ECM accumulation was a classical characteristic feature of tumors, and a higher ECM content predicted a poorer prognosis in a broad range of cancer types [53]. The TME is a composition of cancer cells, non-cancerous stromal cells, soluble growth factors, cytokines, proteases, and ECM, which provides essential signals for tumor survival, growth, and acquisition of invasiveness, while hindering antitumor immunity [24, 54, 55]. Fibroblasts constitute one of the most vital cells in the stroma and turn into cancer-associated fibroblasts (CAFs) in TME. CAFs not only play active roles in tumorigenesis and progression both by soluble factors and direct cell-to-cell contact, but also sculpt TME by suppressing anti-tumor immune responses or by recruiting immunosuppressive cells [54]. Matrisome defined as the compendium of genes encoding core ECM proteins, or structural component of the ECM [53]. Consistently, our results indicated that risk score was positively correlated with the CAF signature, ECM signature, and Matrisome signatures. Moreover, risk score had a positive correlation with stromal score and a negative correlation with tumor purity, which could be on behalf of higher infiltration degrees of stromal cells in the TME of the high-risk group.

The immune cells play essential roles in TME. The success of cancer immunotherapy relies on the comprehensive understanding of the tumor microenvironment and immune evasion mechanisms in which the tumor, stroma, and infiltrating immune cells coordinated in a complex network. The main benefit of immunotherapy is to generate memory CD8^+^ T cells for sustained protection against metastasis and preventing recurrence of the disease [56]. Active immune cells could enter into the tumor parenchyma and perform their anti-tumor function [55]. Therefore, the ultimate goal of immunotherapy is to convert an immunodorminant TME into an immunostimulatory TME, which allows the immune system to clear tumor lesions [56]. Treg cells are involved in tumor progression by inhibiting antitumor immunity. High Treg cell infiltration in the TME was involved in unfavorable prognosis in patients with various types of cancer [53]. M2-polarized macrophages, commonly deemed tumor-associated macrophages (TAMs), were contributors to many pro-tumorigenic outcomes in cancer [57]. Macrophage type 2 (M2) cells, and Tregs cells could make immunologic barriers against CD8^+^ T cell‐mediated antitumor immune responses [58]. Our results indicated that naive B cells, regulatory T cell (Tregs), M0 macrophage and M2 macrophage were significantly enriched in high-risk group, whereas CD8^+^ T cells and resting CD4^+^ T cells were remarkably enriched in low-risk group. Taken together, these results suggested that high-risk group was suffused with immunosuppressive cells such as Tregs, M2 macrophages, producing the immunosuppressive microenvironment to hamper CD8^+^ T cells-mediated eradication for tumor cells.

Monoclonal antibodies against immunological checkpoint molecules provided a vast breakthrough in cancer therapeutics. For instance, PD-1/PD-L1 and CTLA-4 inhibitors showed promising therapeutic outcomes [59]. In our study, high-risk group had a considerably lower rate of immunotherapy response than that in the low-risk group, which was consistent with the expression levels of PD-1/PD-L1 and CTLA-4 in the high-risk and low-risk groups. Interestingly, combination of risk score and immune score, or stromal score can more accurately predict the responsiveness to immunotherapy of patients with STAD. These findings further demonstrated the effectiveness of the risk score as a biomarker in predicating the response to immunotherapy. The TMB was associated with the formation of neoantigens which activated antitumor immunity, which was a reliable biomarker to predict the response to PD-L1 therapy [60]. In our study, the TMB in high-risk group was lower than that in low-risk group, which strongly suggested that the lower response rate to immunotherapy in high-risk group might be due to the lower TMB.

In addition, risk score might be helpful in screening the therapeutics drugs for patients with STAD. For instance, an independent study showed that high-risk STAD patients showed higher sensitivity to the chemotherapy agents, including rapamycin [61]. Another study found that methotrexate is suitable to inhibit the function of Early B-cell factors (EBFs) in gastric cancer [62]. In the present study, rapamycin, PD-0325901 and dasatinib were found to be more effective for patients in the high-risk group, whereas AZD7762, CEP-701 and methotrexate were predicted to be more effective for patients in the low-risk group. However, the toxicities of the screened drugs was uncertain. For instance, due to the unpredictable cardiac toxicity, the development of AZD7762 was not going forward in patients with advanced solid tumors [63]. 5-fluorouracil, doxorubicin, high-dose methotrexate (FAMTX) schedule was reported to be active in advanced gastric cancer, and the main toxicity was myelosuppression [64]. Oxidative stress is a component of many diseases, including cancer. Although numerous small molecule drugs evaluated as antioxidants have exhibited potential therapeutic ability in preclinical studies, results from clinical trial was disappointed. A greater understanding of the pharmacological mechanisms through which anti oxidative drug act might provide a rational usage would lead to greater therapeutic success in malignancies [65, 66].

Our research has some unique advantages. First, the combination of multigene has robust predictive capability for cancer prognosis than single genes. An integrated mitochondria-related gene prognostic risk model would play more vital roles in the diagnosis and prognosis of STAD patients. Second, the results of the study provide us with a more accessible method to determine whether patients belong to the high- or low-risk group, which is simple and feasible. In addition, we evaluated its predictive value, chemotherapy efficacy, immunotherapy efficacy and immune cell infiltration for patients with STAD, which could provide individualized management and treatment for STAD patients.

## Limitations

Although our findings in this study have important clinical consequences, there are still some limitations. Firstly, this is a retrospective study, and an independent prospective cohort is needed to verify the risk model constructed in this study. Secondly, this study heavily relied on datasets and computational predictions while validation component is poor. More experimental studies are needed to validate in further studies. Finally, the carcinogenic effects of the prognostic genes in the model and the mechanisms of interaction between prognostic genes and mitochondrial dysfunction in STAD are mainly unknown and need to be further explored. Based on above information, our future direction will focus on three aspects: (1) Applying mouse model to verify our current hypothesis; (2) Collecting gastric cancer cases and clinical information to validate the risk score model, and compare it with the gold standard of clinical diagnosis, and ensure that the risk model constructed in the present study can be applicable for clinical practice; (3) To screen more novel genes associated with mitochondria in more datasets.

## Conclusions

We established a STAD patient risk score model including NOX4, FKBP10, ALDH3A2, and MAOA. Functionally, the risk score was highly correlated to the TME and immune cell infiltration of STAD patients. Combined analysis for risk score and stromal score, or immune score, or MSS/MSI can predict the response to immunotherapy more accurately than single index in STAD. Regarding drug sensitivity, patients in high-risk group was more sensitive to rapamycin, PD-0325901 and dasatinib, whereas patients in low-risk group was more sensitive to AZD7762, CEP-701 and methotrexate. Taken together, our mitochondrial-related risk model could be a reliable prognostic biomarker for personalized treatment of STAD patients.

## Supplementary Information

**Additional file 1: Figure S1.** Identification of DEGs related to mitochondrion and functional enrichment analysis in STAD. **A** The GO analysis of 183 mitochondrial-related DEGs, including biological process (BP), cellular component (CC), and molecular function (MF).** B** The KEGG analysis of 183 mitochondrial-related DEGs.** C** Protein expressions of the 3 prognosis-related genes in gastric cancer tissues and normal gastric tissues from HPA database. **D** Kaplan–Meier curves for OS of patients with high or low expression of each prognosis-related genes.

**Additional file 2: Figure S2.** Assessing the performance of the prognostic risk model in the validation cohort. **A** Kaplan–Meier curves of the overall survival (OS) in the validation cohort GSE15459.** B** ROC curves for 1-, 3-, and 5-year OS of the prognostic risk model in the validation cohort GSE15459. **C-D** Distribution of risk score, survival status (red dots indicate dead, blue dots indicate alive) and the four genes expression heat map in the validation cohort GSE15459. **E** Gene expression of 4 prognosis-related genes in GSE66229. P values were showed as: ns not significant; *p < 0.05; **p < 0.01; ***p < 0.001

**Additional file 3: Figure S3.** The relationships between the risk score and clinical characteristics of STAD patients. Age, Gender, T stage, N stage, M stage, Tumor stage, Family history of stomach cancer, H pylori infection, Grade, Reflux, Disease type. NX, lymph nodal status could not be determined; MX, metastatic status could not be determined; GX, tumor grade could not be determined; N/A, not available. P values were showed as: ns not significant; *p < 0.05; **p < 0.01; ***p < 0.001

**Additional file 4: Figure S4.** Construction of nomogram using TCGA-STAD cohort. **A** A nomogram was constructed based on risk score and related clinical characteristics.** B** ROC curves and AUC for 1-, 3-, and 5-year OS of the nomogram. **C** Calibration curves of 1-, 3-, and 5-year OS in the nomogram and ideal model. **D** DCA results of risk score and clinical characteristics in the TCGA cohort.

**Additional file 5: Figure S5.** Results of GSEA analysis in low-risk and high-risk groups in STAD. **A** The GSEA findings of the c2 reference gene sets for high-risk groups. **B** The GSEA findings of the c2 reference gene sets for low-risk groups.

**Additional file 6: Figure S6.** The condition of Immune infiltration in low-risk and high-risk group. **A** The proportion of 22 immune cells quantified by CIBERSORT algorithm in low-risk group. **B** The proportion of 22 immune cells quantified by CIBERSORT algorithm in high-risk group. **C** Correlation analysis between the activated CD8 + T cell signatures and CAF signatures, as well as that between the activated CD8 + T cell signatures and matrisome signatures. P values were showed as: ns not significant; *p < 0.05; **p < 0.01; ***p < 0.001

**Additional file 7: Figure S7.** Correlation analysis for risk score and TIDE score in STAD.

**Additional file 8: Figure S8.** Correlation analysis for risk score and 14 immune cells signatures in STAD.

**Additional file 9: Figure S9.** Risk score predicts drug therapeutic benefits in STAD. Proportion of normalized IC50 value of the 138 drugs between the low-risk and high-risk groups. P values were showed as: ns not significant; *p < 0.05; **p < 0.01; ***p < 0.001

**Additional file 10: Tables S1–S14.** **Table S1**. The list of mitochondrial-related genes. **Table S2**. The list of carcinoma associated fibroblast up signatures (n=24). **Table S3**. The list of carcinoma associated fibroblast down signatures (n=24). **Table S4**. The list of ECM and Collagen signatures (n=225). **Table S5**. The list of matrisome signatures (1026). **Table S6**. The primer sequences. **Table S7**. The list of all DEGs in tumor and normal groups (n=2381). **Table S8**. The list of protein-coding DEGs in tumor and normal groups (n=2145). **Table S9**. The list of mitochondrial-related DEGs (n=183). **Table S10**. GO terms in tumor and normal groups. **Table S11**. KEGG terms in tumor and normal groups. **Table 12**. The list of DEGs in high and low risk groups (n=298). **Table S13**. GO terms in high and low risk groups. **Table S14**. KEGG terms in high and low risk groups .

## Data Availability

All data are available in a public, open access repository. R and other custom scripts for analyzing data are available upon reasonable request.

## Abbreviations

STAD: Stomach adenocarcinoma
TME: Tumor microenvironment
GC: Gastric cancer
EBV: Epstein–Barr virus
OS: Overall survival
ROS: Reactive oxygen species
ICB: Immune checkpoint blockade
TMB: Tumor mutation burden
GSEA: Gene set enrichment analyses
DEGs: Differentially expressed genes
K-M: Kaplan–Meier
C-index: Concordance index
HPA: The Human Protein Atlas
NCBI: National Center for Biotechnology Information
DCA: Decision curve analysis
GO: Gene Ontology
KEGG: Kyoto Encyclopedia of Genes and Genomes
TIICs: Tumor-infiltrating immune cells
IC50: 50% Inhibiting concentration
GDSC: Genomics of Drug Sensitivity in Cancer
TIDE: Tumor immune dysfunction and exclusion
ECM: Extracellular matrix
CAF: Carcinoma associated fibroblast
Tregs: Regulatory T cell
MSS: Microsatellite stability
MSI-L: Microsatellite instability-low
MSI-H: Microsatellite instability-high
HER2: Human epidermal growth factor receptor 2
PD-L1: Programmed death-ligand 1
TAMs: Tumor-associated macrophages
M2: Macrophage type 2
BP: Biological process
CC: Cellular component
MF: Molecular function

## References

[1] Sung H, Ferlay J, Siegel RL, Laversanne M, Soerjomataram I, Jemal A, Bray F (2021). Global cancer statistics 2020 GLOBOCAN estimates of incidence and mortality worldwide for 36 cancers in 185 countries. CA Cancer J Clin.

[2] Hoft SG, Noto CN, DiPaolo RJ (2021). Two distinct etiologies of gastric cancer: infection and autoimmunity. Front Cell Dev Biol.

[3] Machlowska J, Baj J, Sitarz M, Maciejewski R, Sitarz R (2020). Gastric cancer: epidemiology, risk factors, classification, genomic characteristics and treatment strategies. Int J Mol Sci.

[4] Poorolajal J, Moradi L, Mohammadi Y, Cheraghi Z, Gohari-Ensaf F (2020). Risk factors for stomach cancer: a systematic review and meta-analysis. Epidemiol Health.

[5] Ilic M, Ilic I (2022). Epidemiology of stomach cancer. World J Gastroenterol.

[6] Tanprasert P, Limpakan S, Chattipakorn SC, Chattipakorn N, Shinlapawittayatorn K (2022). Targeting mitochondria as a therapeutic anti-gastric cancer approach. Apoptosis.

[7] Lee H (2014). Somatic alterations in mitochondrial DNA and mitochondrial dysfunction in gastric cancer progression. World J Gastroenterol.

[8] Rodrigues T, Ferraz LS (2020). Therapeutic potential of targeting mitochondrial dynamics in cancer. Biochem Pharmacol.

[9] Ahmadian E, Babaei H, Mohajjel NA, Eftekhari A, Eghbal MA (2016). Venlafaxine-induced cytotoxicity towards isolated rat hepatocytes involves oxidative stress and mitochondrial/lysosomal dysfunction. Adv Pharm Bull.

[10] Chodari L, Dilsiz AM, Vahedi P, Alipour M, Vahed SZ, Khatibi S, Ahmadian E, Ardalan M, Eftekhari A (2021). Targeting mitochondrial biogenesis with polyphenol compounds. Oxid Med Cell Longev.

[11] Ma X, Gong N, Zhong L, Sun J, Liang XJ (2016). Future of nanotherapeutics: targeting the cellular sub-organelles. Biomaterials.

[12] Li Z, Liu ZM, Xu BH (2018). A meta-analysis of the effect of microRNA-34a on the progression and prognosis of gastric cancer. Eur Rev Med Pharmacol Sci.

[13] Chang W (2014). Inflammation-related factors predicting prognosis of gastric cancer. World J Gastroenterol.

[14] Shao W, Yang Z, Fu Y, Zheng L, Liu F, Chai L, Jia J (2021). The pyroptosis-related signature predicts prognosis and indicates immune microenvironment infiltration in gastric cancer. Front Cell Dev Biol.

[15] Peng Y, Liu C, Li M, Li W, Zhang M, Jiang X, Chang Y, Liu L, Wang F, Zhao Q (2021). Identification of a prognostic and therapeutic immune signature associated with hepatocellular carcinoma. Cancer Cell Int.

[16] Khalaf K, Hana D, Chou JT, Singh C, Mackiewicz A, Kaczmarek M (2021). Aspects of the tumor microenvironment involved in immune resistance and drug resistance. Front Immunol.

[17] Petitprez F, Meylan M, de Reyniès A, Sautès-Fridman C, Fridman WH (2020). The tumor microenvironment in the response to immune checkpoint blockade therapies. Front Immunol..

[18] Samstein RM, Lee CH, Shoushtari AN, Hellmann MD, Shen R, Janjigian YY, Barron DA, Zehir A, Jordan EJ, Omuro A (2019). Tumor mutational load predicts survival after immunotherapy across multiple cancer types. Nat Genet.

[19] Goldman MJ, Craft B, Hastie M, Repečka K, McDade F, Kamath A, Banerjee A, Luo Y, Rogers D, Brooks AN (2020). Visualizing and interpreting cancer genomics data via the Xena platform. Nat Biotechnol.

[20] Rath S, Sharma R, Gupta R, Ast T, Chan C, Durham TJ, Goodman RP, Grabarek Z, Haas ME, Hung W (2021). MitoCarta3.0: an updated mitochondrial proteome now with sub-organelle localization and pathway annotations. Nucleic Acids Res..

[21] Subramanian A, Tamayo P, Mootha VK, Mukherjee S, Ebert BL, Gillette MA, Paulovich A, Pomeroy SL, Golub TR, Lander ES (2005). Gene set enrichment analysis: a knowledge-based approach for interpreting genome-wide expression profiles. Proc Natl Acad Sci U S A.

[22] Mootha VK, Lindgren CM, Eriksson KF, Subramanian A, Sihag S, Lehar J, Puigserver P, Carlsson E, Ridderstrale M, Laurila E (2003). PGC-1alpha-responsive genes involved in oxidative phosphorylation are coordinately downregulated in human diabetes. Nat Genet.

[23] Zhao HB, Zeng YR, Han ZD, Zhuo YJ, Liang YK, Hon CT, Wan S, Wu S, Dahl D, Zhong WD (2021). Novel immune-related signature for risk stratification and prognosis in prostatic adenocarcinoma. Cancer Sci.

[24] Wu J, Li L, Zhang H, Zhao Y, Zhang H, Wu S, Xu B (2021). A risk model developed based on tumor microenvironment predicts overall survival and associates with tumor immunity of patients with lung adenocarcinoma. Oncogene.

[25] Ru B, Wong CN, Tong Y, Zhong JY, Zhong S, Wu WC, Chu KC, Wong CY, Lau CY, Chen I (2019). TISIDB: an integrated repository portal for tumor-immune system interactions. Bioinformatics.

[26] Cerami E, Gao J, Dogrusoz U, Gross BE, Sumer SO, Aksoy BA, Jacobsen A, Byrne CJ, Heuer ML, Larsson E (2012). The cBio cancer genomics portal: an open platform for exploring multidimensional cancer genomics data. Cancer Discov.

[27] Gao J, Aksoy BA, Dogrusoz U, Dresdner G, Gross B, Sumer SO, Sun Y, Jacobsen A, Sinha R, Larsson E (2013). Integrative analysis of complex cancer genomics and clinical profiles using the cBioPortal. Sci Signal.

[28] Gong Z, Zhang J, Guo W (2020). Tumor purity as a prognosis and immunotherapy relevant feature in gastric cancer. Cancer Med.

[29] Lin Y, Pan X, Zhao L, Yang C, Zhang Z, Wang B, Gao Z, Jiang K, Ye Y, Wang S (2021). Immune cell infiltration signatures identified molecular subtypes and underlying mechanisms in gastric cancer. Npj Genom Med.

[30] Chao J, Fuchs CS, Shitara K, Tabernero J, Muro K, Van Cutsem E, Bang YJ, De Vita F, Landers G, Yen CJ (2021). Assessment of pembrolizumab therapy for the treatment of microsatellite instability-high gastric or gastroesophageal junction cancer among patients in the KEYNOTE-059, KEYNOTE-061, and KEYNOTE-062 clinical trials. Jama Oncol.

[31] Martincorena I, Campbell PJ (2015). Somatic mutation in cancer and normal cells. Science.

[32] Marcus L, Fashoyin-Aje LA, Donoghue M, Yuan M, Rodriguez L, Gallagher PS, Philip R, Ghosh S, Theoret MR, Beaver JA (2021). FDA approval summary: pembrolizumab for the treatment of tumor mutational burden-high solid tumors. Clin Cancer Res.

[33] FDA approves first drug for cancers with a high tumor mutational burden. American Cancer Society. 2020.

[34] Kim JY, Kronbichler A, Eisenhut M, Hong SH, van der Vliet HJ, Kang J, Shin JI, Gamerith G (2019). Tumor mutational burden and efficacy of immune checkpoint inhibitors: a systematic review and meta-analysis. Cancers.

[35] Owada-Ozaki Y, Muto S, Takagi H, Inoue T, Watanabe Y, Fukuhara M, Yamaura T, Okabe N, Matsumura Y, Hasegawa T (2018). Prognostic impact of tumor mutation burden in patients with completely resected non-small cell lung cancer: brief report. J Thorac Oncol.

[36] Riviere P, Goodman AM, Okamura R, Barkauskas DA, Whitchurch TJ, Lee S, Khalid N, Collier R, Mareboina M, Frampton GM (2020). High tumor mutational burden correlates with longer survival in immunotherapy-naïve patients with diverse cancers. Mol Cancer Ther.

[37] Choi S, Park S, Kim H, Kang SY, Ahn S, Kim K (2022). Gastric cancer: mechanisms, biomarkers, and therapeutic approaches. Biomedicines.

[38] Roth KG, Mambetsariev I, Kulkarni P, Salgia R (2020). The mitochondrion as an emerging therapeutic target in cancer. Trends Mol Med.

[39] Zong WX, Rabinowitz JD, White E (2016). Mitochondria and cancer. Mol Cell.

[40] Bi Y, Lei X, Chai N, Linghu E (2021). NOX4: a potential therapeutic target for pancreatic cancer and its mechanism. J Transl Med..

[41] Lin X, Yang L, Fu S, Lin W, Gao Y, Chen H, Ge Z (2017). Overexpression of NOX4 predicts poor prognosis and promotes tumor progression in human colorectal cancer. Oncotarget.

[42] Zhang J, Li H, Wu Q, Chen Y, Deng Y, Yang Z, Zhang L, Liu B (2019). Tumoral NOX4 recruits M2 tumor-associated macrophages via ROS/PI3K signaling-dependent various cytokine production to promote NSCLC growth. Redox Biol.

[43] Ramadori G, Ioris RM, Villanyi Z, Firnkes R, Panasenko OO, Allen G, Konstantinidou G, Aras E, Brenachot X, Biscotti T (2020). FKBP10 regulates protein translation to sustain lung cancer growth. Cell Rep.

[44] Gong LB, Zhang C, Yu RX, Li C, Fan YB, Liu YP, Qu XJ (2020). FKBP10 acts as a new biomarker for prognosis and lymph node metastasis of gastric cancer by bioinformatics analysis and in vitro experiments. Onco Targets Ther.

[45] Liang L, Zhao K, Zhu JH, Chen G, Qin XG, Chen JQ (2019). Comprehensive evaluation of FKBP10 expression and its prognostic potential in gastric cancer. Oncol Rep.

[46] Yin Z, Wu D, Shi J, Wei X, Jin N, Lu X, Ren X (2020). Identification of ALDH3A2 as a novel prognostic biomarker in gastric adenocarcinoma using integrated bioinformatics analysis. Bmc Cancer..

[47] Zhao Y, Tao Z, Chen X (2020). A three-metabolic-genes risk score model predicts overall survival in clear cell renal cell carcinoma patients. Front Oncol.

[48] Chen L, Guo L, Sun Z, Yang G, Guo J, Chen K, Xiao R, Yang X, Sheng L (2020). Monoamine oxidase A is a major mediator of mitochondrial homeostasis and glycolysis in gastric cancer progression. Cancer Manag Res.

[49] Liao C, Lin T, Li P, Geary LA, Chen K, Vaikari VP, Wu JB, Lin C, Gross ME, Shih JC (2018). Loss of MAOA in epithelia inhibits adenocarcinoma development, cell proliferation and cancer stem cells in prostate. Oncogene.

[50] Tang CT, Gao YJ, Ge ZZ (2018). NOX4, a new genetic target for anti-cancer therapy in digestive system cancer. J Dig Dis.

[51] Nie Y, Liu L, Liu Q, Zhu X (2021). Identification of a metabolic-related gene signature predicting the overall survival for patients with stomach adenocarcinoma. PeerJ.

[52] Wu M, Xia Y, Wang Y, Fan F, Li X, Song J, Ding J (2020). Development and validation of an immune-related gene prognostic model for stomach adenocarcinoma. Bioscience Rep..

[53] Izzi V, Davis MN, Naba A (2020). Pan-cancer analysis of the genomic alterations and mutations of the matrisome. Cancers.

[54] Gok Yavuz B, Gunaydin G, Kosemehmetoglu K, Karakoc D, Ozgur F, Guc D (2018). The effects of cancer-associated fibroblasts obtained from atypical ductal hyperplasia on anti-tumor immune responses. Breast J.

[55] Labani-Motlagh A, Ashja-Mahdavi M, Loskog A (2020). The tumor microenvironment: a milieu hindering and obstructing antitumor immune responses. Front Immunol..

[56] Locy H, de Mey S, de Mey W, De Ridder M, Thielemans K, Maenhout SK (2018). Immunomodulation of the tumor microenvironment: turn foe into friend. Front Immunol..

[57] Boutilier AJ, Elsawa SF (2021). Macrophage polarization states in the tumor microenvironment. Int J Mol Sci.

[58] Farhood B, Najafi M, Mortezaee K (2019). CD8(+) cytotoxic T lymphocytes in cancer immunotherapy: a review. J Cell Physiol.

[59] Darvin P, Toor SM, Sasidharan Nair V, Elkord E (2018). Immune checkpoint inhibitors: recent progress and potential biomarkers. Exp Mol Med.

[60] Xu F, Huang X, Li Y, Chen Y, Lin L (2021). m6A-related lncRNAs are potential biomarkers for predicting prognoses and immune responses in patients with LUAD. Mol Ther Nucleic Acids.

[61] Tong X, Yang X, Tong X, Zhai D, Liu Y (2022). Complement system-related genes in stomach adenocarcinoma: Prognostic signature, immune landscape, and drug resistance. Front Genet.

[62] Wang Q, Liang J, Hu X, Gu S, Xu Q, Yan J (2021). Early B-cell factors involve in the tumorigenesis and predict the overall survival of gastric cancer. Biosci Rep..

[63] Sausville E, Lorusso P, Carducci M, Carter J, Quinn MF, Malburg L, Azad N, Cosgrove D, Knight R, Barker P (2014). Phase I dose-escalation study of AZD7762, a checkpoint kinase inhibitor, in combination with gemcitabine in US patients with advanced solid tumors. Cancer Chemother Pharmacol.

[64] Bar SG, Tsalic M, Gaitini D, Steiner M, Haim N (2002). Etoposide, doxorubicin and cisplatin alternating with 5-fluorouracil, doxorubicin and high-dose methotrexate in patients with advanced adenocarcinoma of the stomach or the gastroesophageal junction. J Chemother.

[65] Forman HJ, Zhang H (2021). Author correction: targeting oxidative stress in disease: promise and limitations of antioxidant therapy. Nat Rev Drug Discov.

[66] Ahmadian E, Eftekhari A, Kavetskyy T, Khosroushahi AY, Turksoy VA, Khalilov R (2020). Effects of quercetin loaded nanostructured lipid carriers on the paraquat-induced toxicity in human lymphocytes. Pestic Biochem Physiol.

