# Supplementary figures and images for: Constructing a novel mitochondrial-related gene signature for evaluating the tumor immune microenvironment and predicting survival in stomach adenocarcinoma
Source: J Transl Med. 2023 Mar 13;21:191. doi: 10.1186/s12967-023-04033-6 (PMC10012538; doi:10.1186/s12967-023-04033-6)

## Figure S1

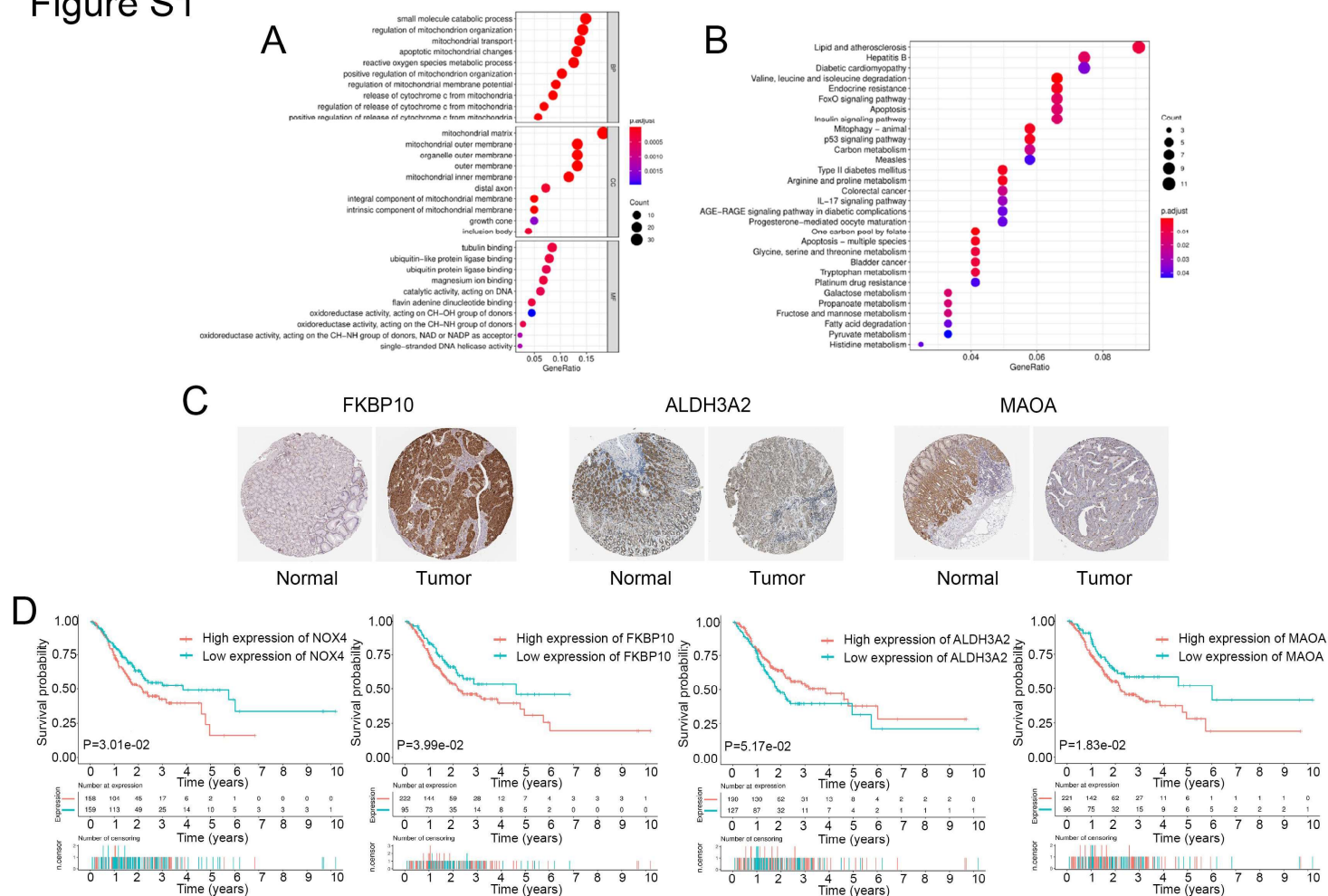

Supplement: Supplementary file 1 — Additional file 1: Figure S1. Identification of DEGs related to mitochondrion and functional enrichment analysis in STAD. A The GO analysis of 183 mitochondrial-related DEGs, including biological process (BP), cellular component (CC), and molecular function (MF). B The KEGG analysis of 183 mitochondrial-related DEGs. C Protein expressions of the 3 prognosis-related genes in gastric cancer tissues and normal gastric tissues from HPA database. D Kaplan–Meier curves for OS of patients with high or low expression of each prognosis-related genes. [file 12967_2023_4033_MOESM1_ESM.pdf]

Figure S2

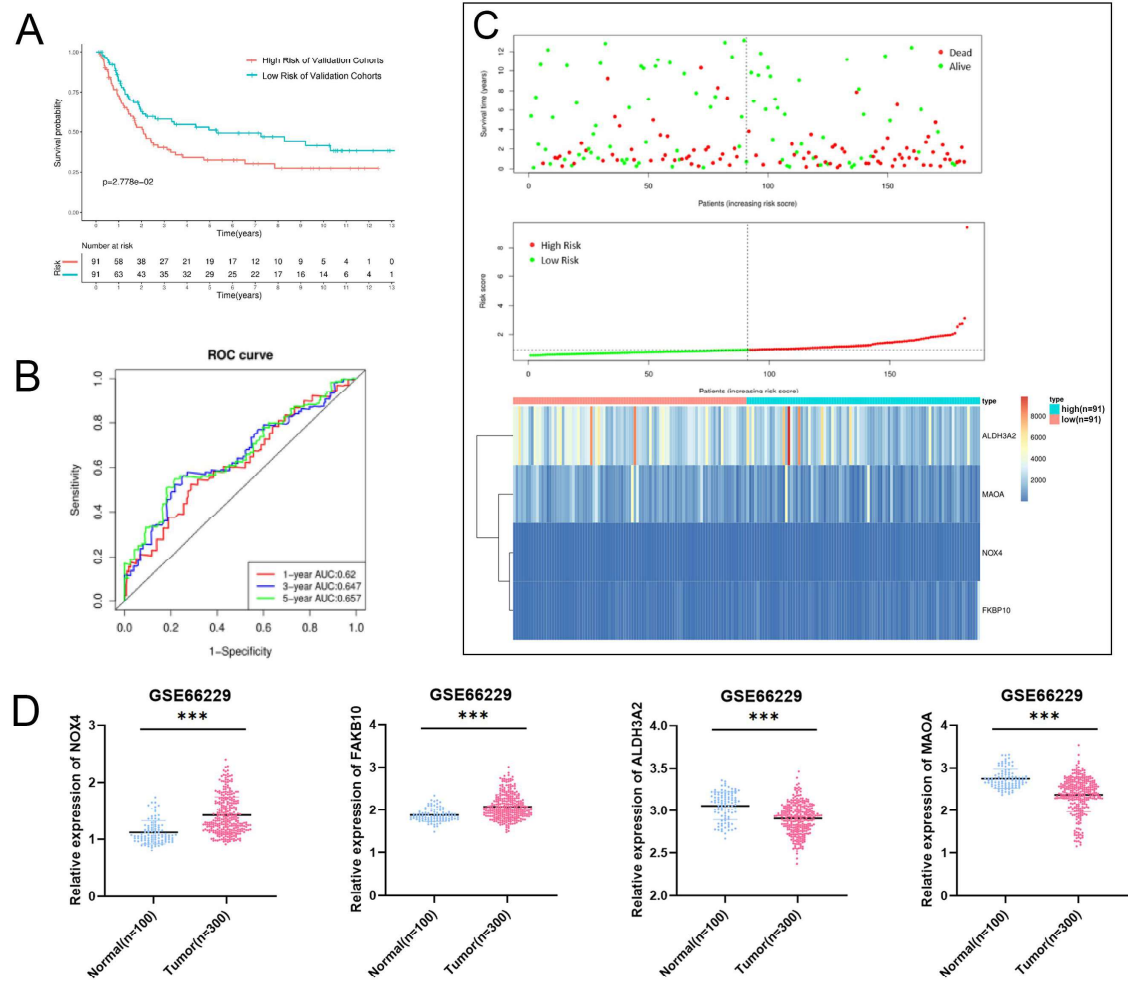

Supplement: Supplementary file 2 — Additional file 2: Figure S2. Assessing the performance of the prognostic risk model in the validation cohort. A Kaplan–Meier curves of the overall survival (OS) in the validation cohort GSE15459. B ROC curves for 1-, 3-, and 5-year OS of the prognostic risk model in the validation cohort GSE15459. C-D Distribution of risk score, survival status (red dots indicate dead, blue dots indicate alive) and the four genes expression heat map in the validation cohort GSE15459. E Gene expression of 4 prognosis-related genes in GSE66229. P values were showed as: ns not significant; *p < 0.05; **p < 0.01; ***p < 0.001 [file 12967_2023_4033_MOESM2_ESM.pdf]

Figure S3

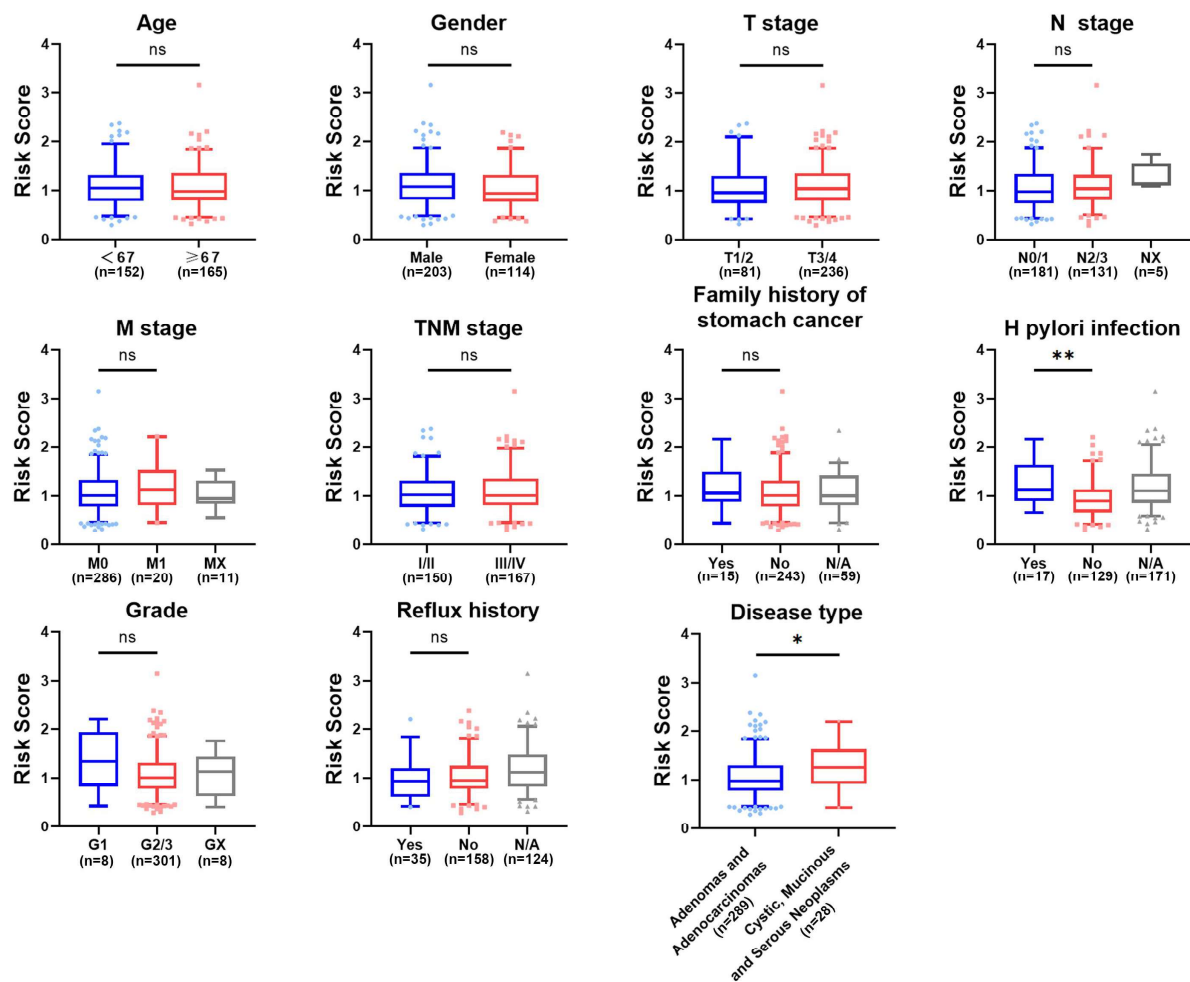

Supplement: Supplementary file 3 — Additional file 3: Figure S3. The relationships between the risk score and clinical characteristics of STAD patients. Age, Gender, T stage, N stage, M stage, Tumor stage, Family history of stomach cancer, H pylori infection, Grade, Reflux, Disease type. NX, lymph nodal status could not be determined; MX, metastatic status could not be determined; GX, tumor grade could not be determined; N/A, not available. P values were showed as: ns not significant; *p < 0.05; **p < 0.01; ***p < 0.001 [file 12967_2023_4033_MOESM3_ESM.pdf]

Figure S4

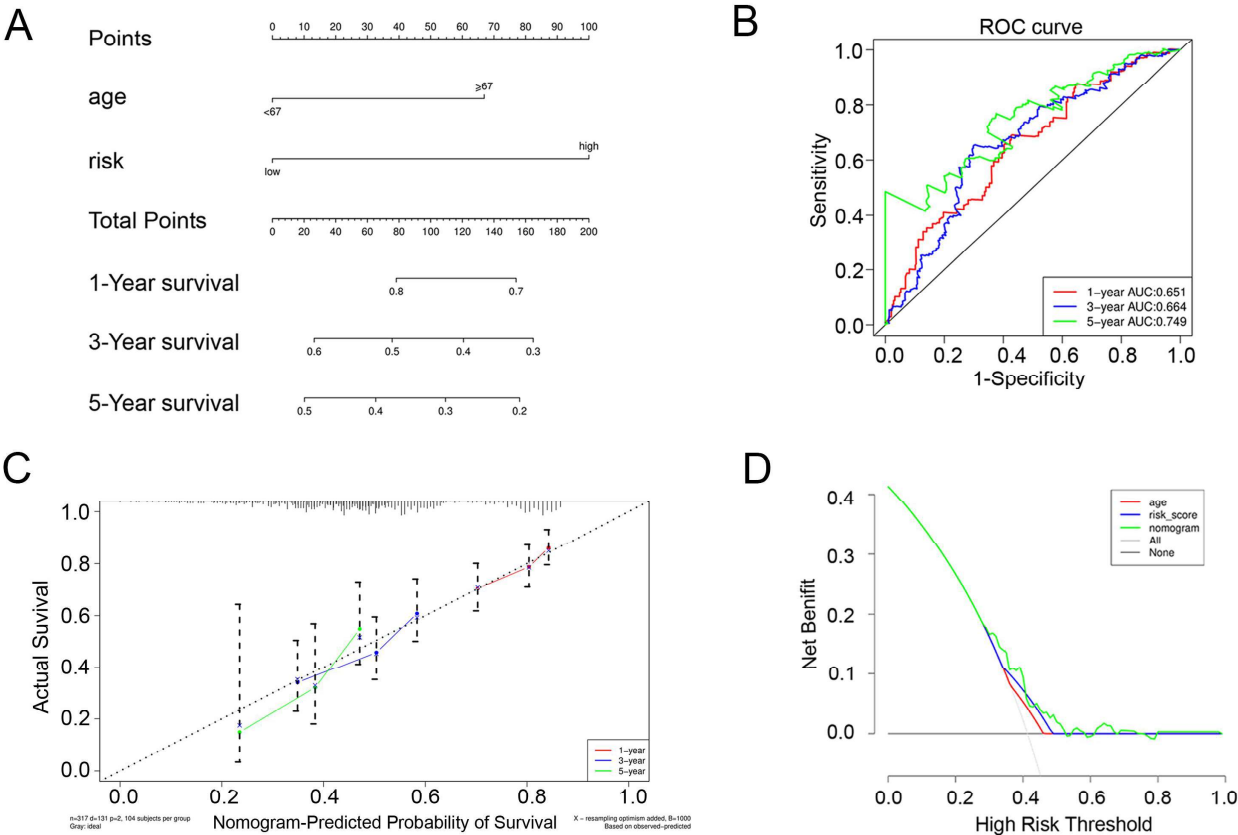

Supplement: Supplementary file 4 — Additional file 4: Figure S4. Construction of nomogram using TCGA-STAD cohort. A A nomogram was constructed based on risk score and related clinical characteristics. B ROC curves and AUC for 1-, 3-, and 5-year OS of the nomogram. C Calibration curves of 1-, 3-, and 5-year OS in the nomogram and ideal model. D DCA results of risk score and clinical characteristics in the TCGA cohort. [file 12967_2023_4033_MOESM4_ESM.pdf]

Figure S5

A

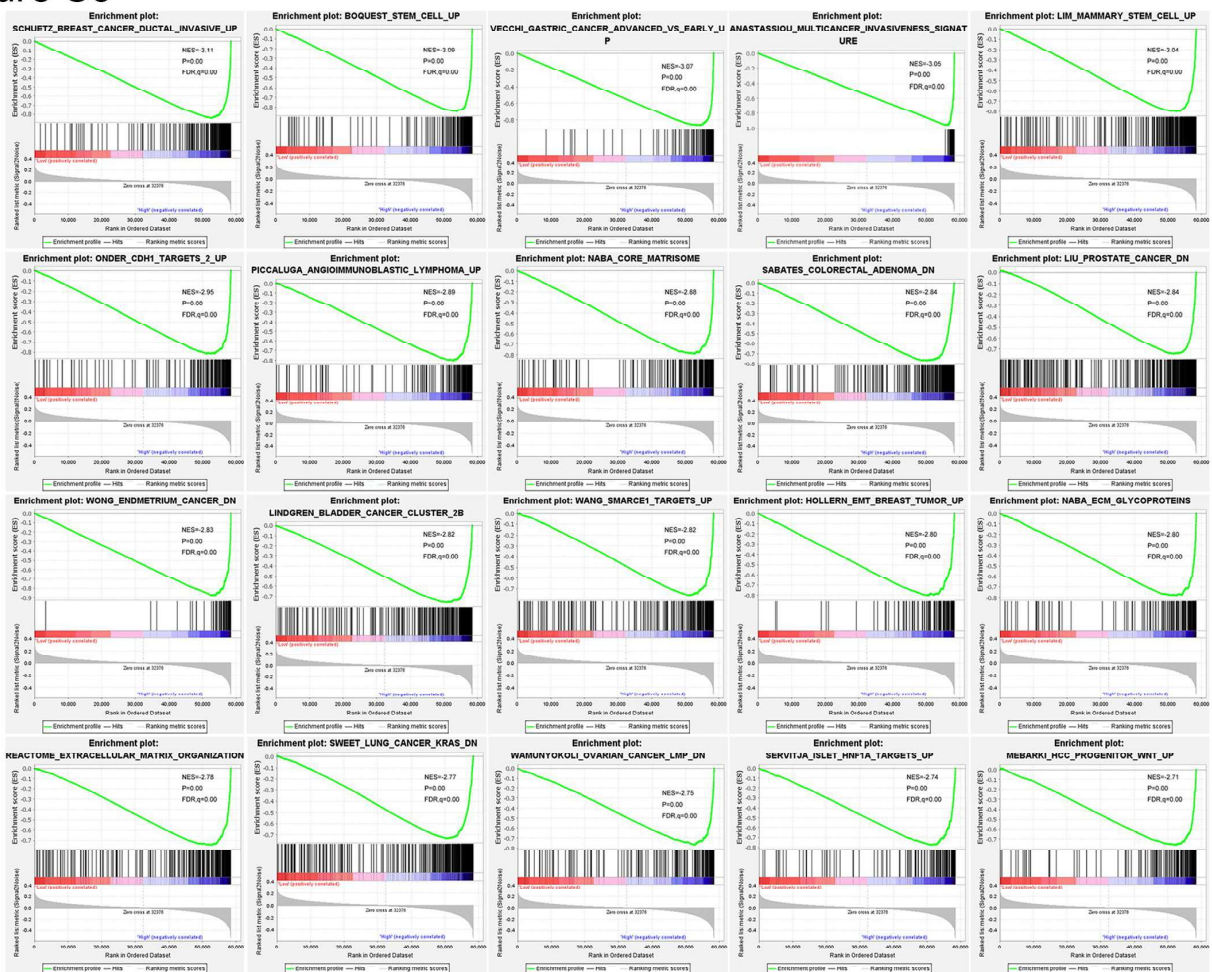

B

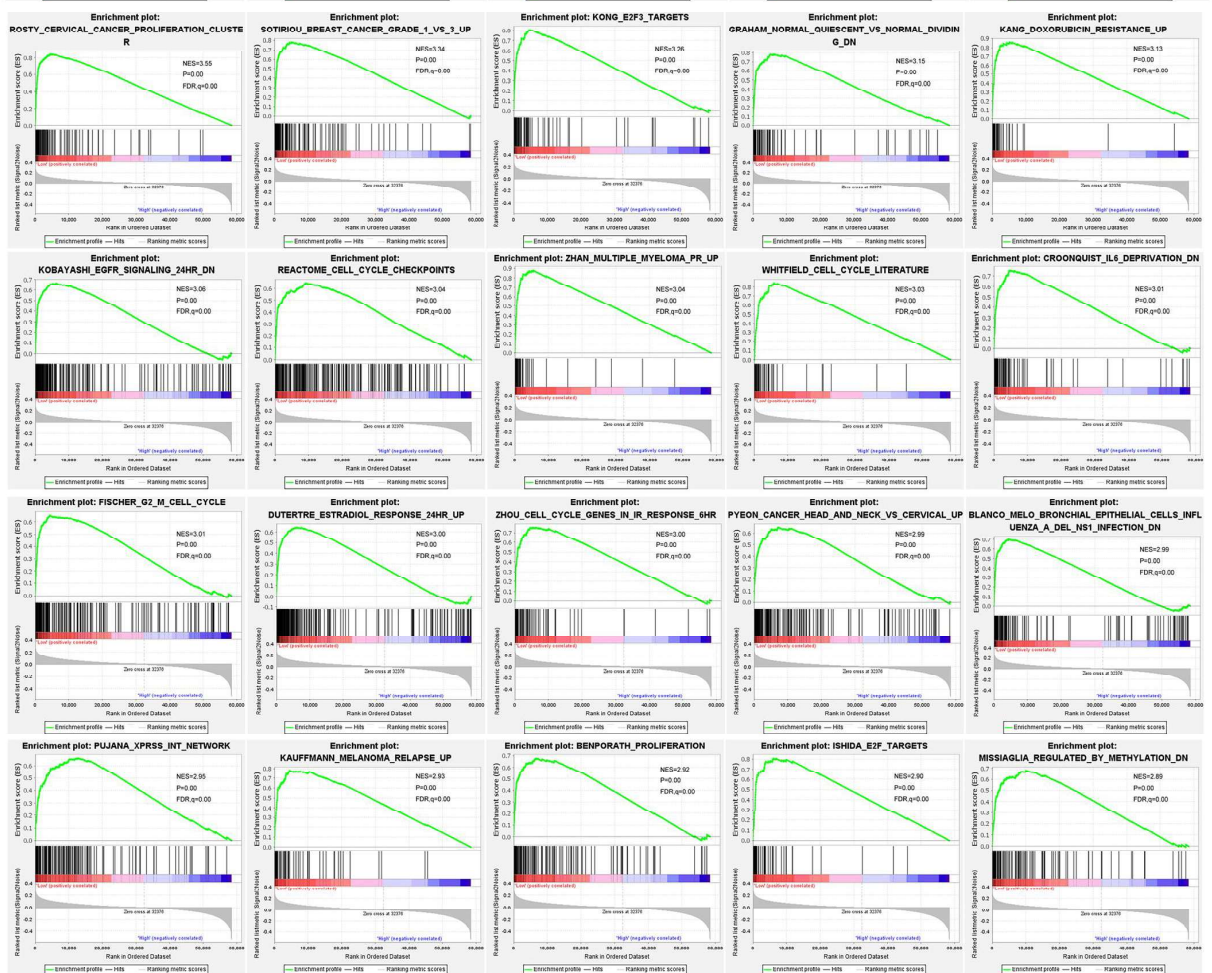

Supplement: Supplementary file 5 — Additional file 5: Figure S5. Results of GSEA analysis in low-risk and high-risk groups in STAD. A The GSEA findings of the c2 reference gene sets for high-risk groups. B The GSEA findings of the c2 reference gene sets for low-risk groups. [file 12967_2023_4033_MOESM5_ESM.pdf]

Figure S6

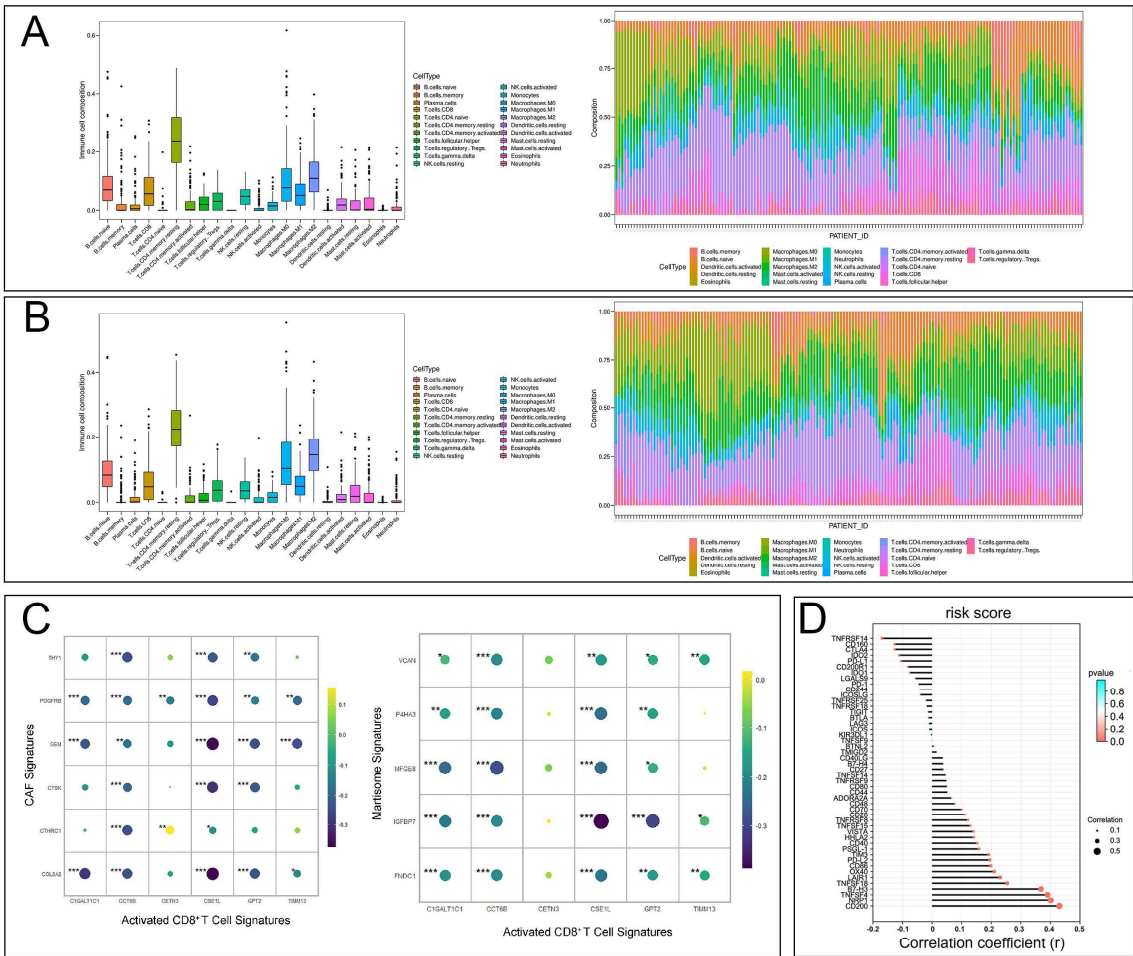

Supplement: Supplementary file 6 — Additional file 6: Figure S6. The condition of Immune infiltration in low-risk and high-risk group. A The proportion of 22 immune cells quantified by CIBERSORT algorithm in low-risk group. B The proportion of 22 immune cells quantified by CIBERSORT algorithm in high-risk group. C Correlation analysis between the activated CD8 + T cell signatures and CAF signatures, as well as that between the activated CD8 + T cell signatures and matrisome signatures. P values were showed as: ns not significant; *p < 0.05; **p < 0.01; ***p < 0.001 [file 12967_2023_4033_MOESM6_ESM.pdf]

Figure S7

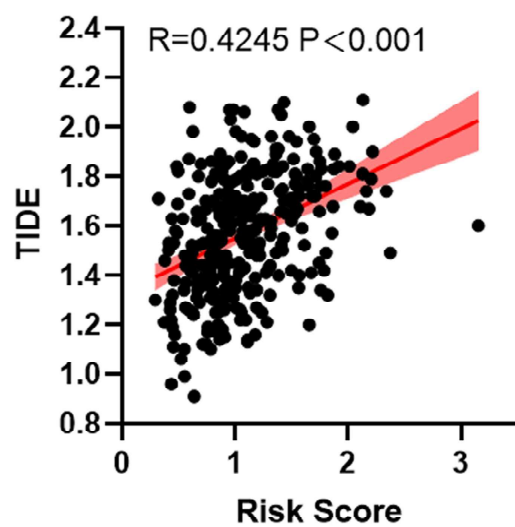

Supplement: Supplementary file 7 — Additional file 7: Figure S7. Correlation analysis for risk score and TIDE score in STAD. [file 12967_2023_4033_MOESM7_ESM.pdf]

# Figure S8

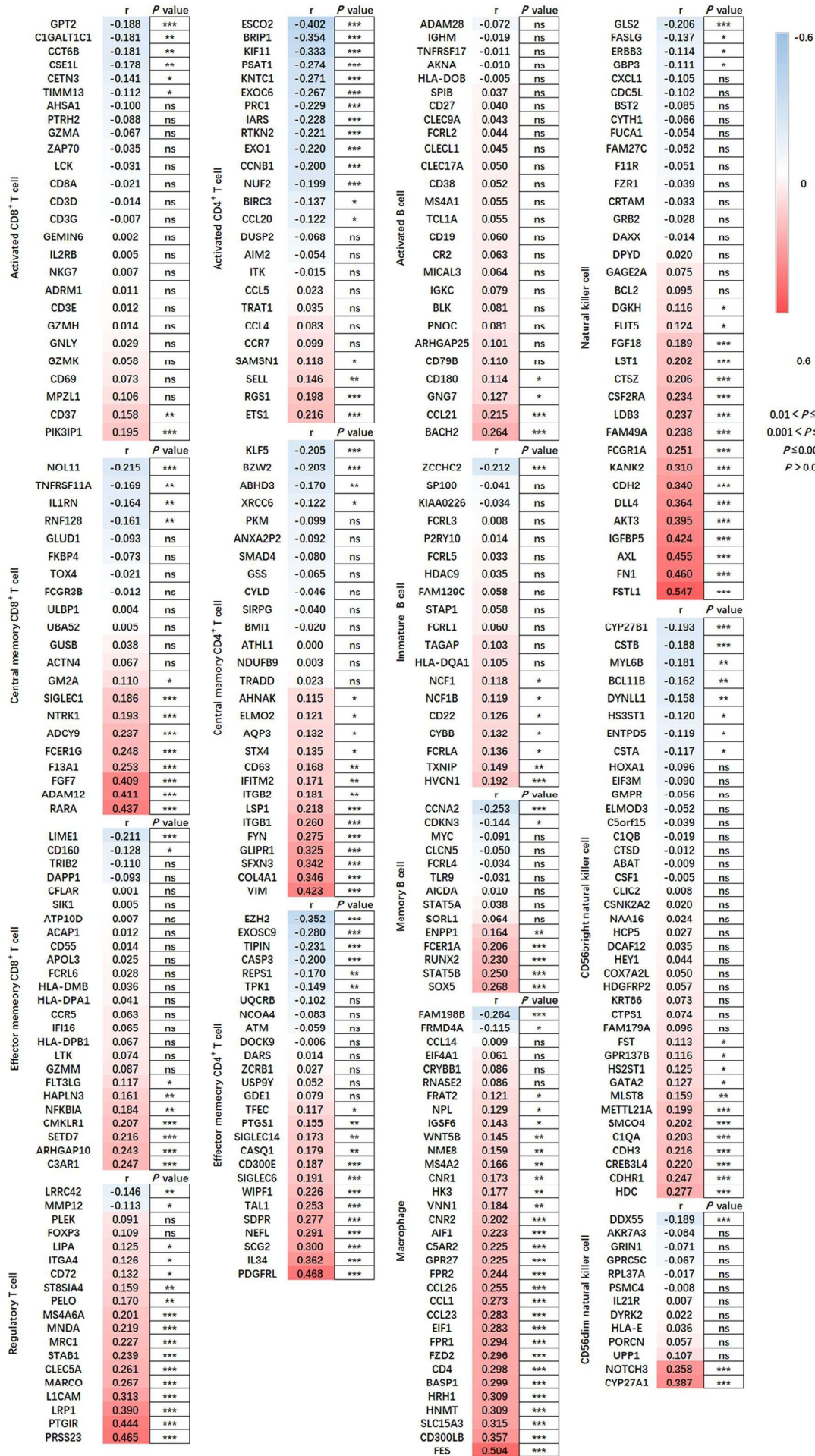

Supplement: Supplementary file 8 — Additional file 8: Figure S8. Correlation analysis for risk score and 14 immune cells signatures in STAD. [file 12967_2023_4033_MOESM8_ESM.pdf]

Figure S9

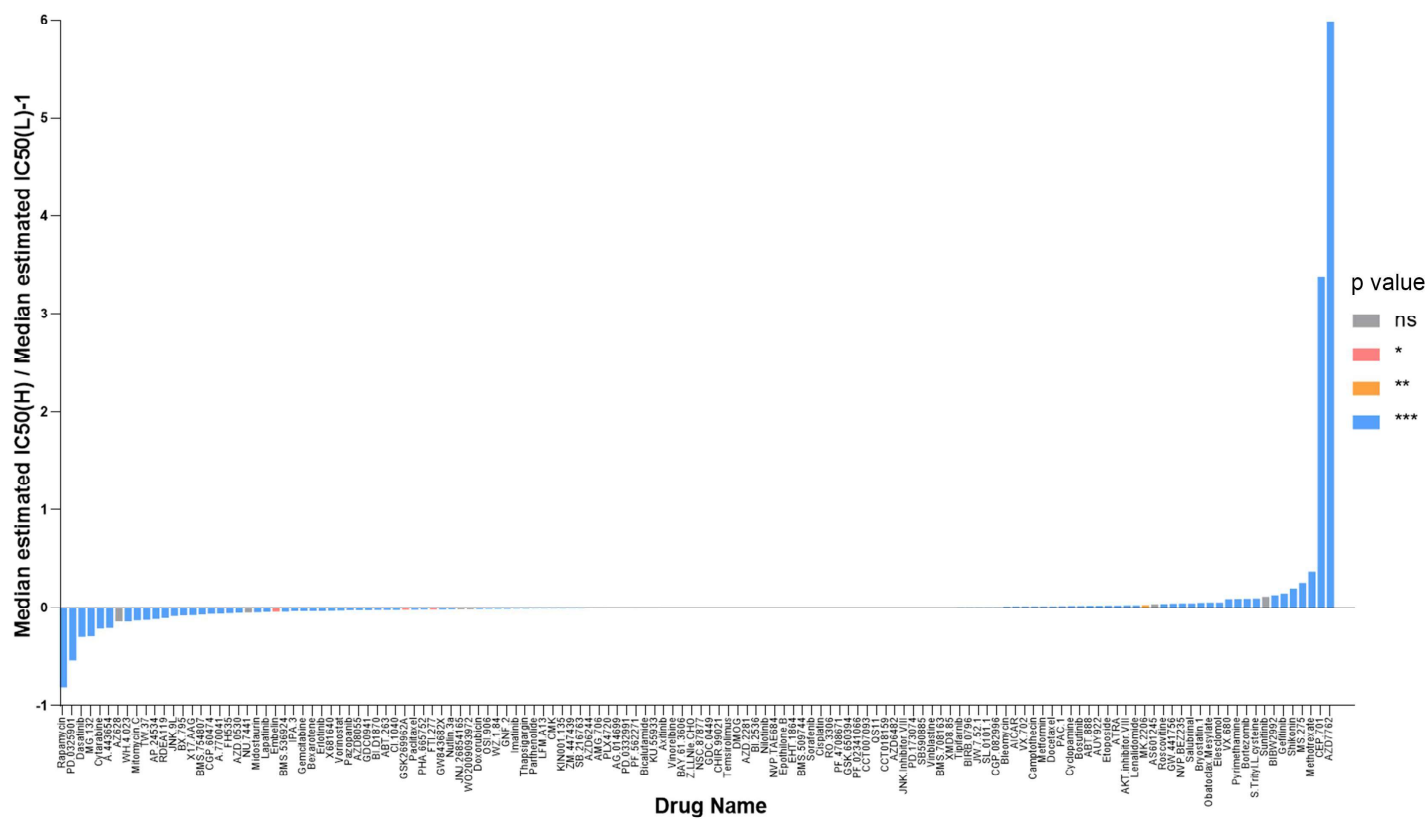

Supplement: Supplementary file 9 — Additional file 9: Figure S9. Risk score predicts drug therapeutic benefits in STAD. Proportion of normalized IC50 value of the 138 drugs between the low-risk and high-risk groups. P values were showed as: ns not significant; *p < 0.05; **p < 0.01; ***p < 0.001 [file 12967_2023_4033_MOESM9_ESM.pdf]
